# The replicative fitness and virulence of potato virus Y evolve differently in pepper lines with different levels of resistance and tolerance

**DOI:** 10.1099/jgv.0.002208

**Published:** 2026-02-02

**Authors:** Thibaud Jayet, Lucie Tamisier, Marion Szadkowski, Elise Lepage, Grégory Girardot, Loup Rimbaud, Véronique Lefebvre, Benoît Moury

**Affiliations:** 1 Pathologie Végétale, INRAE, 84140 Montfavet, France; 2 GAFL, INRAE, 84140 Montfavet, France

**Keywords:** bottleneck, *Capsicum annuum*, experimental evolution, malevolence, potato virus Y (PVY), *Potyvirus yituberosi*, resistance durability

## Abstract

Despite their great agronomic interest and widespread occurrence in germplasm resources, the quantitative resistance and tolerance of plants to their parasites have rarely been studied in terms of durability potential. Using experimental evolution under controlled conditions for 9 months, we compared the evolution of potato virus Y (PVY) (*Potyvirus yituberosi*) virulence, measured by the effect of viral infection on plant fresh weight, and replicative fitness, measured by systemic viral load, in five pepper (*Capsicum annuum*) lines contrasting in their levels of quantitative resistance and tolerance. PVY evolutionary trajectories differed between pepper lines. Three lines revealed either an increase in PVY replicative fitness or an increase or decrease in PVY virulence. Two other lines did not reveal any significant change in PVY replicative fitness or virulence. The tolerance level of three pepper lines also differed significantly when measured with initial and evolved PVY populations, often associated with changes in PVY virulence. PVY evolutionary trajectories were partly explained by parameters linked to plant resistance operating at different stages of infection (inoculation, colonization of inoculated leaves and systemic infection). This study provides information on the durability potential of quantitative resistance and tolerance to PVY in pepper.

## Introduction

Breeding for resistant crops is an important lever for the management of viral diseases and epidemics in the field, firstly because there are no chemical treatments against viruses and secondly because resistant cultivars have no harmful impact on the environment or human health [1,3]. Depending on their level of efficacy, we can distinguish between total and quantitative (or partial) resistance, which reduces the pathogen load in the plant almost totally or only partially, respectively. The first type of resistance is generally determined by a major-effect resistance gene, while the second is generally determined by quantitative trait loci (QTLs).

The high evolutionary potential of viruses leads to frequent ‘resistance breakdowns’, i.e. the partial or total abolition of resistance efficacy, resulting in major losses in plant production. Consequently, studies have been conducted to determine the factors that increase the durability potential of resistance, focusing mainly on major-effect resistance genes [4,8]. In contrast, little empirical data are available on the durability potential of quantitative resistance to viruses or other pathogens. A common assumption is that we can expect a higher durability potential for quantitative resistance than for total resistance, because the former only partially reduces pathogen multiplication and, therefore, induces lower selection pressure on pathogen populations. However, there is experimental evidence of increased infectivity, virulence (i.e. the quantitative impact of parasite infection on host health or fitness) or load of plant parasites faced with quantitative host resistance. In their comprehensive review, Cowger and Brown [9] cited 21 studies that demonstrated the selection of increased pathogen virulence by quantitative plant resistance. Only two of these studies concerned viruses, showing their adaptation to plant resistance [10,11]. There could, however, be a potential publication bias, as experiments that revealed no change in pathogen virulence or no adaptation to host plants are less likely to be published.

In addition to resistance, tolerance is also considered a major agronomic trait for managing viral disease epidemics. While plant resistance reduces parasite load, plant tolerance reduces the damage caused by a parasite without necessarily impacting its load in the plant [12,13]. Several studies have focused on the genetic determinism of virus tolerance in plants (e.g. 14,17) or on ways to estimate tolerance (reviewed in [18]). It should be noted that tolerance can be defined qualitatively or quantitatively. Qualitative tolerance corresponds to the absence (or low level) of plant damage despite infection, while intolerance corresponds to a high level of damage in plants with the same level of infection. The problem with this definition is that, in most cases, damage and infection levels vary simultaneously between plant genotypes, making it difficult to distinguish between tolerance and quantitative resistance. Therefore, in this study, we used a quantitative definition of tolerance, also named ‘range tolerance’, corresponding to the slope of the linear regression between plant damage and viral load [18,19]. The greater the increase in plant damage per unit of viral load, the lower the tolerance of the plant genotype.

Despite these advances in understanding and measuring tolerance, its durability potential is largely unknown. In the well-studied *Zym* gene conferring zucchini (*Cucurbita pepo*) tolerance to zucchini yellow mosaic virus (ZYMV) (*Potyvirus cucurbitaflavitesselati*, genus *Potyvirus*, family Potyviridae), a viral mutation responsible for a loss of tolerance has been characterized [20]. This mutation has also resulted in a competitive cost for the virus during infection of zucchini varieties lacking the *Zym* gene, and this cost can be exploited to reduce the impact of the disease.

The general lack of knowledge about the durability potential of quantitative resistance and tolerance in plants led us to evaluate them using the potato virus Y (PVY) – *Capsicum annuum* (pepper) system. To this end, we first carried out experimental evolution (EE) of PVY variants on pepper lines showing contrasting levels of resistance and tolerance. Next, we compared the systemic viral loads and impact on plant weight of PVY initial variants and final populations. Finally, we used parameters linked to the dynamics of PVY infection in the different pepper lines to interpret the evolutionary trajectories of PVY populations.

## Methods

### Plant and virus material

As host plants, we chose the five pepper lines HD223, HD253, HD2334, HD2341 and HD2397 (*C. annuum* L., family *Solanaceae*) that showed contrasting levels of quantitative resistance and tolerance to PVY in previous experiments [16,21, 22]. Indeed, we measured a fourfold difference in PVY load at the systemic level between the most (HD223) and least resistant (HD253) lines (Table S1, available in the online Supplementary Material). Regarding tolerance, symptom observations suggested that four lines were rather tolerant to PVY infection, showing only systemic mosaics, while the last one (HD2397) was intolerant and showed systemic necroses [16]. However, we have not quantitatively estimated the tolerance levels of these lines (using range tolerance) [19]. These lines are part of a doubled-haploid (DH) pepper progeny and are perfectly homozygous [21]. Consequently, the plant replicates are genetically identical for a given DH line. The five lines selected do not carry the major-effect resistance gene *pvr2^3^*, which segregates in this progeny.

Two PVY variants (*Potyvirus yituberosi*, genus *Potyvirus*, family Potyviridae) derived from the SON41p clone were used [23]. The single mutant SON41p-115K (hereafter K) carries a lysine substitution at amino acid position 115 of VPg, while the double mutant SON41p-101G-115K (hereafter GK) carries a glycine substitution at amino acid position 101 of VPg in addition to the lysine at position 115. We have used these variants in previous evolution experiments [24,25]. In preliminary experiments, we observed that variant K was slightly more competitive than variant GK in the five pepper lines used in this study (unpublished data). However, in the present study, variants K and GK were not significantly different in terms of their ability to replicate within the plants in the absence of competition. Prior to the EEs, we multiplied both variants by mechanical inoculation of 2-week-old *Nicotiana benthamiana* seedlings, in order to obtain high-titre inocula. For each variant, 1 g of virus-infected dehydrated leaves was ground with 4 ml of phosphate buffer (0.03 M Na_2_HPO_4_, 0.2% sodium diethyldithiocarbamate), 90 mg activated charcoal and 90 mg carborundum using pestles and mortars. Leaves from infected *N. benthamiana* plants were harvested 20 days post-inoculation (dpi) and used to prepare inocula for the first infection cycle of pepper plants, which constituted the initial variants of the EE.

### Experimental evolution

For the EE, we inoculated all five pepper lines with PVY variant K, while only three lines (HD223, HD2334 and HD2397) were inoculated with variant GK, given our experimental capacity to perform the EE on up to 64 virus lineages (8 plant–virus combinations × 8 plant replicas). Plants were arranged in a fully randomized design in a climatic chamber (photoperiod 14 h day/10 h night, 24 °C/21 °C). We inoculated the first two leaves of ten 3-week-old seedlings from each DH pepper line with PVY. Twenty-six dpi, we harvested 1 gram of fully developed leaves from below the apex of each plant for PVY diagnosis using double-antibody sandwich ELISA (DAS-ELISA). Next, a second infection cycle was initiated 28 days after the start of the first cycle with eight out of the ten plants (when available) that had tested positive in ELISA, selected at random for each combination of initial PVY variant and pepper line, to initiate eight independent evolutionary lineages.

**Fig. 1.**
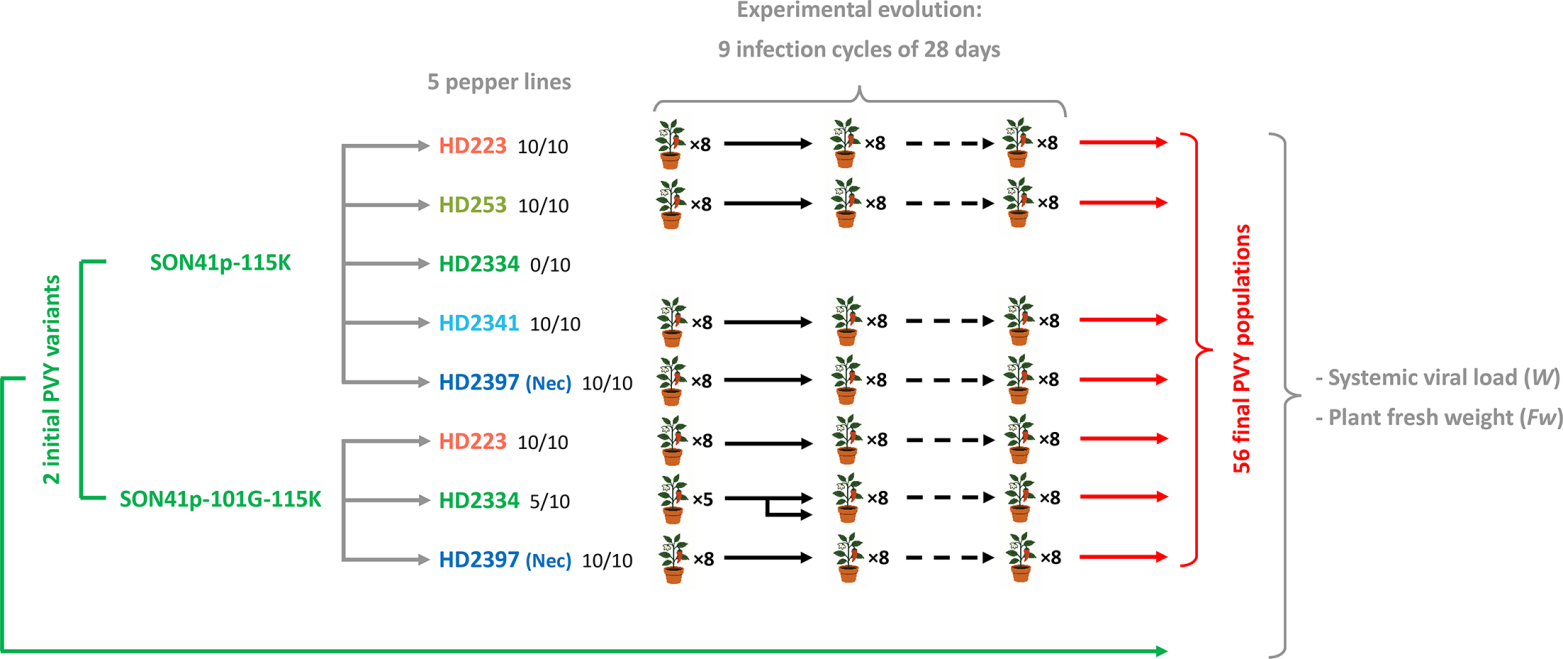
EE design and phenotypic evaluation of initial variants and final populations of PVY. The EE consisted of nine serial infection cycles using two variants of the SON41p infectious clone. The SON41p-115K (K) variant, carrying a lysine substitution at amino acid position 115 of the VPg of SON41p, was serially inoculated on five DH pepper lines, while the SON41p-101G-115K (GK) variant, carrying a glycine substitution at amino acid position 101 of the VPg of SON41p, was serially inoculated only on HD223, HD2334 and HD2397. Each combination of viral variant and DH pepper line was used to create eight independent PVY evolutionary lineages. The number of infected plants among the 10 inoculated during the first infection cycle is indicated next to the names of pepper lines. On HD2334, the EE was interrupted as early as the second cycle for variant K (no infected plants) and only five plants were infected during the first cycle for variant GK. Therefore, to initiate three additional lineages for variant GK on HD2334, infected plants initiating evolutionary lineages 1, 3 and 5 were randomly selected to initiate two lineages each from the second cycle (lineages number 2, 6 and 8, respectively). At the end of the EE, each of the 56 final populations was compared with its initial variant for systemic viral load (*W*), a proxy for PVY replicative fitness, and for plant fresh weight (*Fw*), used to estimate PVY virulence, on the same DH line that was used for the entire EE. *Fw* of ten mock-inoculated plants belonging to each pepper DH line was also measured. A fully randomized design was followed for this experiment. The initial PVY variants are indicated in green and the final populations in red. The colour code of the five pepper lines was also used in the other figures. HD2397 is the only pepper line that shows systemic necrosis (Nec) when infected with PVY.

To initiate the second and subsequent infection cycles, three 3-week-old seedlings were inoculated for each of the evolutionary lineages. For each lineage, one of the three inoculated plants was selected at random to initiate the next cycle. A total of nine consecutive infection cycles were carried out (Fig. 1). The PVY variant–pepper line combinations were named with the name of the initial variant and the number of the DH line (e.g. K223 is the combination between variant K and line HD223).

### Assessment of replicative fitness and virulence of initial variants and final evolved populations of PVY

After the EE, we measured the systemic load of each of the final PVY populations and initial variants on the corresponding pepper line where the virus evolved, as well as the fresh weight of infected plants (Fig. 1). We used the plants infected at the end of the ninth infection cycle to produce inocula corresponding to the final populations. For the two initial PVY variants K and GK, inocula were prepared in each pepper line corresponding to the EE (i.e. seven inocula in total) and in *N. benthamiana* as a backup in case pepper plants were not infected due to their resistance level (i.e. two inocula in total). All final PVY populations and the two initial variants were inoculated onto ten seedlings of the corresponding DH line. PVY load was assessed by quantitative DAS-ELISA relative to a reference viral sample, by collecting and grinding 1 g of fully expanded leaves immediately below the apex per plant (i.e. a pooled sample of two or three leaves), as described in [26]. In addition, we added ten mock-inoculated seedlings per DH line. Along with host range and transmissibility, systemic viral load is one component of viral fitness that corresponds to the ‘replicative fitness’ [27,28], and we will denote it by *W_i_* for initial variants and *W_f_* for final populations. We compared the systemic viral load between final populations and the corresponding initial PVY variant with *ΔW*=*W_f_ –W_i_* and *rW*=*W_f_*/* W_i_*.

PVY virulence was estimated by the effect of viral infection on plant fresh weight. At 28 dpi, we cut the plants at the cotyledon node and weighed them. Previous results have shown that fresh and dry plant weights of DH lines from this pepper progeny were strongly correlated under our test conditions (Pearson correlation *r*=0.957 [10]). For each infected plant, PVY virulence was estimated as $VIR=\frac{\overset{-}{{Fw}_{mock}}-{Fw}_{inf}}{\overset{-}{{Fw}_{mock}}}$, where $\overset{-}{{Fw}_{mock}}$ is the average fresh weight of mock-inoculated plants of a line and *Fw_inf_* is the fresh weight of the infected plant of the same line. Next, *VIR_i_* and *VIR_f_* were used as virulence estimates for initial PVY variants and final populations, respectively, and we compared their virulence with *ΔVIR*=*VIR_f_ –VIR_i_* and *rVIR=VIR_f_*/* VIR_i_*.

### Estimation of the tolerance level of pepper lines to PVY

For each pepper line, we estimated the level of tolerance to PVY infection as the slope of the linear regression between plant biomass, estimated by the fresh weight of infected plants (*Fw*), and systemic viral load (*W*), following the range tolerance method [18,19]. We applied this method in two different ways, including (i) infected plants only or (ii) both infected and mock-inoculated plants (for which *W*=0). *TOL_i_* and *TOL_f_* are estimates of pepper tolerance to initial PVY variants and final populations, respectively, and differences in plant tolerance to final populations and initial variants were estimated as *ΔTOL*=*TOL_f_ TOL_i_*.

### Estimation of parameters characterizing pepper quantitative resistance to colonization of inoculated leaves

To explain the differences in replicative fitness and virulence between the initial PVY variants and the corresponding final populations, we measured several parameters characterizing the level of quantitative resistance of pepper DH lines at the inoculation stage and during colonization of the inoculated leaf by PVY (Fig. S1A). For the inoculation stage, we counted the number of PVY infection foci between 5 and 7 dpi after mechanical inoculation of the first two leaves of ten plants per DH line with a green fluorescent protein (GFP)-tagged version of variant K (Fig. S1E). As inoculum, we used a standardized dose of a PVY suspension at 500 µg µl^−1^ purified following [29]. Tamisier *et al*. [30] and Zwart *et al*. [31] showed that the vast majority of infection foci observed following mechanical inoculation of pepper leaves with GFP-tagged potyviruses were initiated by a single virus particle. Thus, provided the inoculum is standardized, the average number of foci per leaf estimated for each DH line provides an estimate of the size of the bottleneck experienced by the viral population at the inoculation stage. This number will be denoted *N_e_*, as it is a proxy for the PVY effective population size at the inoculation stage.

To characterize the colonization of inoculated leaves, we followed the dynamics of infection with the same GFP-tagged variant. One of the first two leaves of eight seedlings per pepper line was inoculated with purified virus and then cut at the base of the leaf blade at 5 dpi. We stored these leaves in boxes containing moistened blotting paper for a further 17 days and photographed them every 1 to 3 days. Photographs were taken under standardized lighting conditions, under blue light (emission band 460–495 nm, corresponding to the GFP excitation peak) and with a green filter (bandwidth 500–515 nm, corresponding to the GFP emission peak) to follow the expansion of the fluorescent zone (Fig. S1A, S1F). The images were then analysed using a Python script designed for semantic image segmentation into three categories based on the colour of each pixel: background (black), healthy leaf tissue (dark green) and fluorescent leaf tissue (light green) (Fig. S1B). This script is based on a Gaussian mixture model, an unsupervised machine learning algorithm based on the assumption that an image can be considered as a combination of several underlying subpopulations of pixels – three in our case – each characterized by a Gaussian distribution. We jointly estimated the parameters of each Gaussian distribution (mean, covariance and weight in the Gaussian mixture) for all images of a given DH line at a given date. We then used these parameters to assign each pixel to one of the three categories. The percentage of fluorescent leaf area (*P_fluo_*, the ratio of the number of pixels in the fluorescent category to the total number of pixels in the leaf) was thus obtained at nine time points *t* ranging from 5 to 21 dpi. Colonization dynamics were then modelled using Verhulst’s logistic growth model $P_{fluo}(t)=\frac{k}{1+e^{-4s(t-\mu )}}$ (Fig. S1C). Thus, we can describe the colonisation dynamics within each leaf by the three parameters: *µ* (the *x* value at the inflection point of the curve, in dpi), *k* (the *y* value of the asymptote of the logistic curve, i.e. the carrying capacity) and *s* (the slope at the inflection point, after adjustment by *k*). We then calculated the averages of the parameters *µ*, *k* and *s* for each pepper line (Fig. S1D, Table S1).

Unlike *W*, which describes plant resistance to systemic infection, *N_e_* (effective population size at inoculation) describes plant resistance to inoculation, and *µ*, *k* and *s* characterize different components of plant resistance to colonization of the inoculated leaf: delay, intensity and speed of colonization, respectively. Radar diagrams summarizing the susceptibility/resistance profiles of the five DH lines in relation to these five traits are shown in Fig. S2.

### Statistical analyses

We performed all statistical analyses with R software version 4.3.3, except path analyses, which were performed with R version 4.4.3. The versions of the R packages used are shown in Table S2.

We tested whether the PVY evolution (with *ΔW*, *rW*, *ΔVIR* and *rVIR* as response variables) differed significantly between pepper lines with mixed-effects models, where ‘pepper line’ was a fixed effect and ‘virus lineage’ was a random effect nested within the ‘pepper line’ effect, using the package *nlme*. We performed the same analysis with the ‘pepper line–initial variant combination’ as the fixed effect. For the evolution of PVY replicative fitness, the variable *rW* best met the assumptions of residual normality and homoscedasticity, i.e. homogeneity of variances (0.01<*P*<0.05) after applying a reciprocal transformation. For the evolution of PVY virulence, the variable *ΔVIR* best met the assumption of residual normality (*P*=0.011) after applying a log transformation. The Bartlett test revealed a violation of homoscedasticity (*P*=2e−10), but visual inspection revealed no substantial discrepancies in variance.

We compared the systemic viral load and plant fresh weights between each final PVY population and the corresponding initial variant using Dunnett tests with the package *DescTools*. We also compared the plant fresh weights between each final PVY population or initial variant and the mock-inoculated plants of the same pepper line using Dunnett tests. Model assumptions were tested with a Shapiro–Wilk test (normality of residuals) and a Bartlett test (homoscedasticity) and by visual inspection of the distribution of residuals.

To determine whether the tolerance level of a given pepper line differed significantly between final PVY populations and the corresponding initial variant, we compared two linear models obtained with the *lm* function. The null model corresponds to *Fw*~*W*, where *Fw* is the plant fresh weight and *W* the systemic viral load, and considers indifferently plants infected by a given final PVY population or by the corresponding initial variant. The full model corresponds to *Fw*~*W : State*, where *State* is an additional binary variable representing the initial or final state of the virus. In this model, the regression slope is different for the final PVY population and the initial variant, but the intercept is identical. Mock-inoculated plants of the same pepper line were included in both models. As the full and null models are nested, we performed a likelihood ratio test (LRT) with the *lrtest* function of the *lmtest* package to assess whether allowing different slopes for the initial and final PVY results in a significantly better fit to the data. This approach makes it possible to determine whether the tolerance level of the pepper line differs significantly between a given final PVY population and the corresponding initial variant.

We used generalized linear models (GLMs) to analyse the links between the response variables *ΔW*, *W_f_*, *ΔVIR, VIR_f_, ΔTOL* and *TOL_f_* characterizing the evolutionary trajectories observed during PVY EE and the explanatory variables *N_e_*, *µ*, *k*, *s*, *W_i_*, *VIR_i_* and *TOL_i_*. For *ΔW* (and *ΔVIR* and *ΔTOL*, respectively), we did not include *W_i_* (and *VIR_i_* and *TOL_i_*, respectively) among the explanatory variables due to spurious correlation problems associated with non-independence between response and explanatory variables [25,32]. We assumed Gaussian distributions for the GLM residuals, which we tested with Shapiro–Wilk tests.

As several pairs of explanatory variables had relatively high correlation coefficients, we expected problems of multicollinearity in GLMs. Consequently, we first calculated variance inflation factors (VIFs) associated with GLMs comprising only main effects (i.e. without interaction terms). We started with the most complex models and we dropped explanatory variables one by one until there were no multicollinearity issues, i.e. VIFs below 6.0 for all variables. For all GLMs retained after this first stage, we carried out a stepwise model selection using the corrected Akaike information criterion (cAIC), starting with models including all variables and their pairwise interactions. We used packages *car* (VIFs), *MuMIn* (model selection), *ggplot2* and *ggpubr* (data visualization), *pscl* (estimation of R-squared) and *visreg splines* and *rgl* (linear model plots) to perform GLM analyses. We analysed correlations between variables with R packages *corrplot* and *PerformanceAnalytics*.

We performed path analyses [33] with R packages *lavaan* and *semPlot* to further analyse the putative cause and effect relationships between predictor traits associated with pepper resistance (*N_e_*, *μ*, *k* and *s*) and response variables corresponding to PVY evolution (*ΔW*, *ΔVIR* and *ΔTOL*). We analysed three hypothetical path scenarios for each response variable separately and three additional scenarios combining all three response variables. Among these latter, model A hypothesizes that *N_e_* has both a direct and an indirect effect (through accelerating the PVY colonization of inoculated leaves, i.e. reducing *μ*, or increasing its carrying capacity *k*) on *ΔW* and *ΔVIR*, whereas models B and C hypothesize that *N_e_* has only direct or indirect effects on *ΔW* and *ΔVIR*, respectively.

The R Markdown script covering all the statistical analyses performed in this article is available online at https://zenodo.org/records/15720482.

Datasets, including raw and processed data, are available in File S1.

## Results

### Changes in replicative fitness or virulence during the EE of PVY are specific to each pepper line

In total, the EE consisted of 56 evolutionary lineages corresponding to seven combinations between two initial PVY variants and five pepper lines, with eight independent evolutionary lineages per combination. These lineages were propagated by mechanical inoculation during nine successive infection cycles (Fig. 1). Due to the high level of resistance to inoculation of HD2334 (low *N_e_*, Table S2 and Fig. S2), EE of variant K in line HD2334 could not be performed, as all plants were ELISA-negative at the end of the first cycle. For the combination of variant GK and line HD2334, only five of the ten inoculated plants were infected by the end of the first cycle. Consequently, the three missing PVY evolutionary lineages to reach a total of eight were initiated in the second infection cycle, using three plants selected at random from the five that had been infected. For each infection cycle and for each lineage, all inoculated plants showed mosaic and/or necrosis at the systemic level (except in the first cycle for line HD2334 as mentioned above).

Next, we conducted an independent experiment to compare the replicative fitness and virulence of the 56 final PVY populations with their corresponding initial variants in the pepper lines in which they evolved (Fig. 1). For the initial PVY variants, no significant differences were generally observed between inocula prepared from *N. benthamiana* and pepper. In these cases, we retained the results obtained with these two inocula in order to increase the sample size and the power of the statistical analyses. However, inocula of variant K multiplied in pepper plants of HD253 and HD2341 induced significantly lower systemic viral loads than inocula of the same virus multiplied in *N. benthamiana* (data not shown). Consequently, in both cases, results obtained with inocula multiplied in *N. benthamiana* were excluded from further analyses and only results obtained with inoculum multiplied in HD253 and HD2341 were retained. This excluded any bias due to the plant species used to prepare the inocula.

More than 99% of inoculated plants were systemically infected in pepper lines HD253, HD2341 and HD2397. In contrast, infection rates were lower in the lines HD223 and HD2334. In HD223, 34/40 (85%) and 144/160 (90%) plants were infected with initial variants and final populations, respectively, and in HD2334, 15/20 (75%) and 72/80 (90%) plants were infected with initial variants and final populations, respectively. These lower infection rates could be explained by the stronger PVY resistance of these two lines, either at the inoculation stage (HD2334) or at the systemic level (HD223) (see below). We have removed the data corresponding to uninfected plants for the following analyses.

**Fig. 2.**
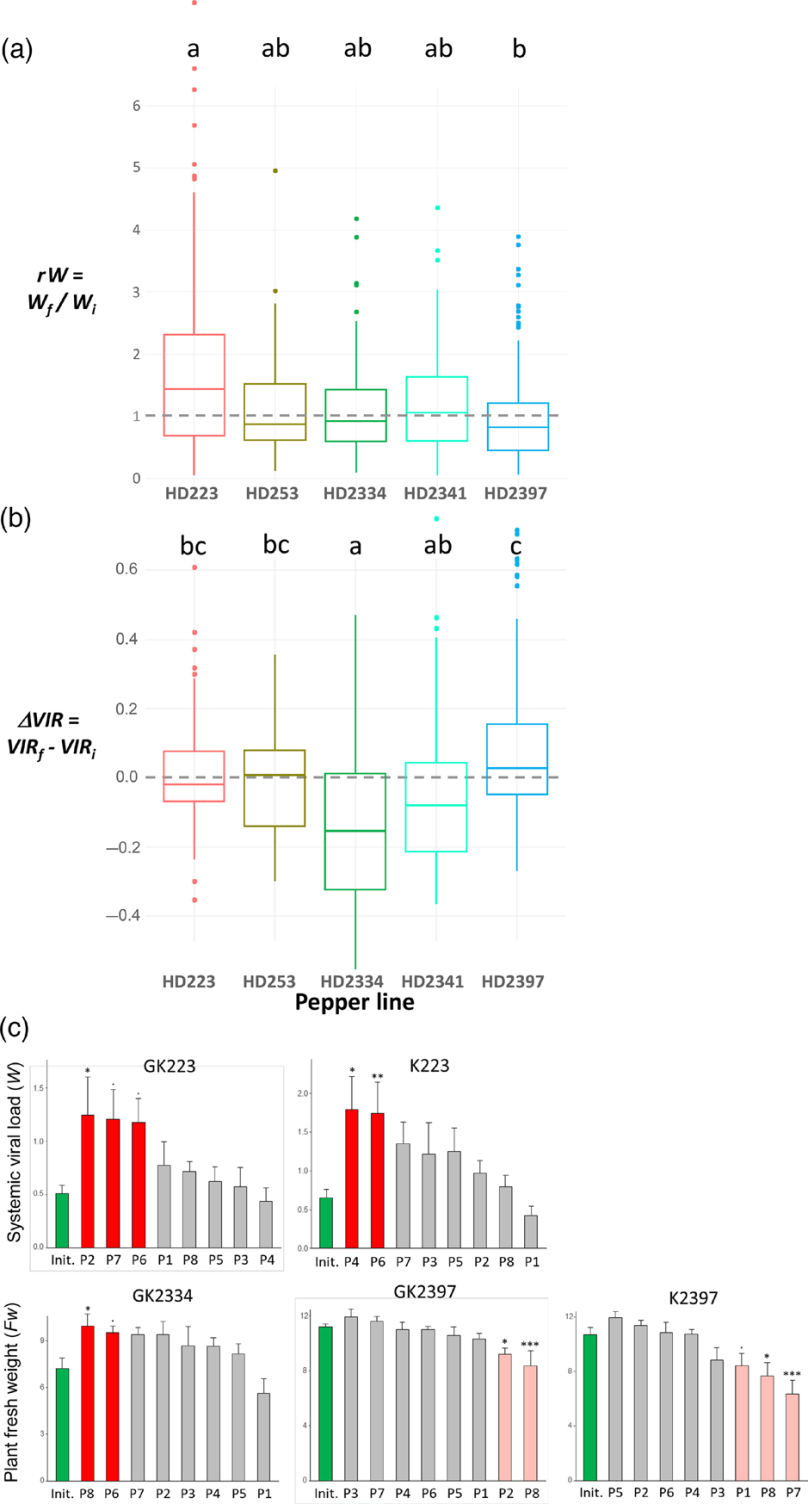
Changes in replicative fitness (*W*; estimated by the systemic viral load) or virulence (*VIR*; estimated by the virus effect on plant fresh weight *Fw*) during PVY EE. (a) Ratio between the systemic viral loads of final populations and corresponding initial variant in five pepper DH lines. Pepper lines sharing the same letter(s) (on the top) are not significantly different based on fitting a mixed-effects model followed by Tukey’s test. The grey dotted line indicates no difference in systemic viral load between the initial PVY variant and final populations (*rW*=1). (b) Difference between the virulences of final populations and corresponding initial variant in five pepper DH lines. Pepper lines sharing the same letter(s) (on the top) are not significantly different based on fitting a mixed-effects model followed by Tukey’s test. The grey dotted line indicates no difference in virulence between the initial PVY variant and final populations (*ΔVIR*=0). (c) Systemic viral load and virulence of initial variants (Init.; green bars) and the eight derived final populations (P1 to P8) for five initial virus variant–pepper line combinations (indicated at the top of each diagram). Asterisks indicate final populations showing a significant (*P<0.05; **P<0.01; ***P<0.001) or marginally significant (·: 0.05<*P*<0.10) difference from the initial variant with the Dunnett test. No significant differences (*P*>0.10) were observed for the three remaining combinations of initial variant and pepper line. Bright red bars: increase in trait value during EE. Light red bars: decrease in trait value during EE. The PVY variants used to initiate the EE were the K and GK variants of the SON41p clone. Error bars represent standard errors.

We then analysed the evolutionary trajectories of PVY by calculating the magnitude of the change in replicative fitness or in virulence. First, we tested the null hypothesis that the evolutionary trajectories did not differ among pepper lines or among combinations of the initial virus and the pepper line. For *rW*, representing the change in replicative fitness, the effect of *pepper line* was significant (*P*=0.0012) and indicated that the increase in replicative fitness was significantly higher for HD223 than for HD2397, with the three other lines showing intermediate values, not significantly different from HD223 or HD2397 (Fig. 2a). A significant effect of *virus lineage* was also detected (*P*=0.002). Similar results were obtained with the explanatory variable *initial virus–pepper line combination*. For HD223, the mean increases in systemic viral load were 84% for variant K and 66% for variant GK.

For *ΔVIR*, representing the change in virulence, the effect of *pepper line* was significant (*P*<0.0001) and showed that the increase in virulence was significantly lower for HD2334 than for HD223, HD253 and HD2397 (Fig. 2b). Virulence also increased significantly less for HD2341 than for HD2397. A significant effect of *virus lineage* was again observed (*P*<0.0001). Similar results were obtained with the explanatory variable *initial virus–pepper line combination*, except that no significant difference in virulence change was detected between K2341 and either K2397 or GK2397 (data not shown).

We then compared each final PVY population individually with its corresponding initial variant.

In HD223, the replicative fitness increased significantly for the two final populations, P4 and P6, derived from variant K and for the population P2 derived from variant GK (Dunnett tests, *P*<0.05). The increase was also marginally significant (0.10<*P*<0.05) for two additional populations (P6 and P7) derived from variant GK (Fig. 2c). The systemic viral load of these five final populations was 130–175% higher than that of the corresponding initial variant. For the other pepper lines, no significant differences in replicative fitness were detected between the final populations and the initial variants.

Regarding PVY virulence, no significant differences were observed between the fresh weights (*Fw*) of mock-inoculated plants and PVY-infected plants of HD223 and HD2341 – either inoculated with the final populations or with the initial variant (Dunnett tests, *P*>0.05). This suggests a high level of tolerance to PVY infection for these two pepper lines, consistent with previous results for HD223 [10]. In contrast, PVY infection significantly reduced *Fw* for at least one of the PVY populations (either the initial variant or a final population) for pepper lines HD253, HD2334 and HD2397 (Dunnett tests, *P*<0.05), indicating lower levels of tolerance. This effect was observed for 7 of the 16 final populations for HD2397, 1 of 8 final populations and the initial variant for HD2334 and 1 of 8 final populations for HD253 (data not shown). For HD2334, PVY virulence decreased significantly for population P8 derived from variant GK (Dunnett test, *P*<0.05) and was marginally lower than that of the initial variant GK (0.10<*P*<0.05) for population P6 (Fig. 2c). For HD2397, four final populations showed higher virulence than the initial variants K or GK (P7 and P8 for variant K and P2 and P8 for variant GK, *P*<0.05; Dunnett tests, Fig. 2c). In addition, final population P1 exhibited a marginally higher virulence than the initial variant K (0.10<*P*<0.05). For the three other pepper lines, no significant differences in virulence were detected between the final populations and the initial variants.

We observed no significant difference in replicative fitness or virulence between the final populations evolved in HD253 or HD2341 and the initial variant K (Dunnett tests). No final PVY population showed simultaneous changes in replicative fitness and virulence. These results were confirmed by the absence of any significant correlation between *ΔVIR* and *ΔW* among the 56 final PVY populations (Pearson correlation coefficient *r*=−0.076; *P*=0.576), suggesting independent evolutionary trajectories.

### Pepper tolerance is significantly altered after PVY evolution

To estimate the range tolerance of pepper DH lines to PVY, the linear regression between plant fresh weight *Fw* and systemic viral load *W* was more appropriate when mock-inoculated plants were included (for which *W*=0) (Table S3), and only results obtained with this method will be shown in the following. HD223 was highly tolerant to PVY infection, with a non-significant correlation between *Fw* and *W* for variant GK or a significantly positive correlation for variant K. We have observed this positive correlation between viral load and plant health, an overcompensation phenomenon [34], previously for pepper line HD223 inoculated with another PVY variant [10]. HD2341 was also tolerant to both variants of PVY, with a non-significant correlation between *Fw* and *W*. The other three pepper lines (HD253, HD2334 and HD2397) were rather intolerant to PVY infection, with significantly negative correlations between *Fw* and *W.* We expected HD2397 to be intolerant, given that it is the only line to show systemic necrosis upon PVY infection, associated with an unfavourable tolerance allele on chromosome 9 (Table S1) [16,21]. The intolerance of HD2334 and HD253 to PVY was more unexpected, since these lines carry the favourable tolerance allele on chromosome 9 (Table S1) and do not show systemic necrosis upon PVY infection.

**Fig. 3.**
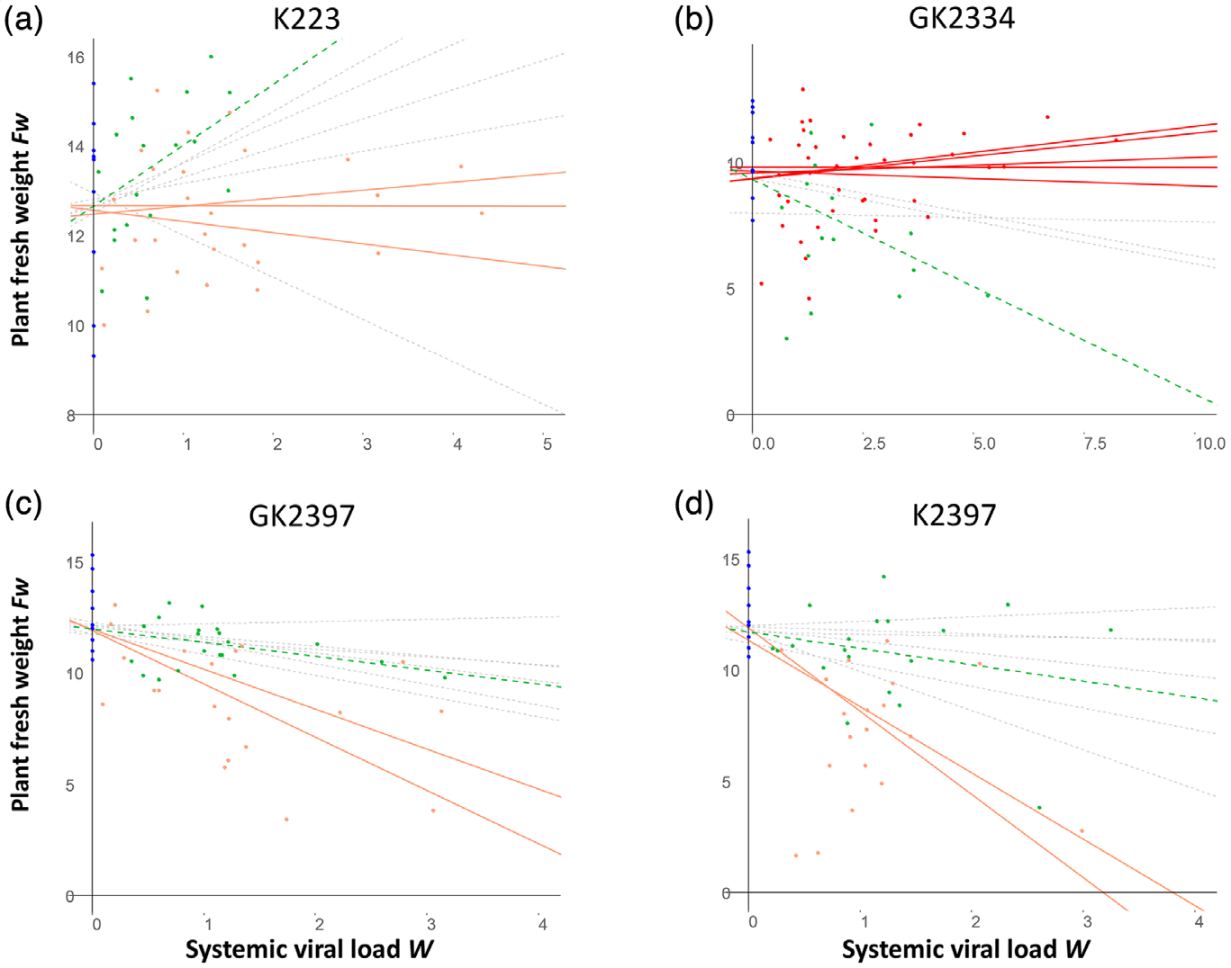
Differences in tolerance of pepper lines inoculated with either final PVY populations or the respective initial variant. We estimated plant tolerance by the slope of the linear regression between plant fresh weight (*Fw*, in grams) and systemic viral load (systemic viral load *W*). Tolerance comparisons were made by comparing the null model *Fw*~*W*, where plants infected by a given final PVY population or by the corresponding initial variant are not differentiated, with the full model *Fw*~*W : State*, where *State* is an additional binary variable representing either the initial variant or the final population of PVY. Only virus–plant combinations with significant tolerance differences based on an LRT (Table S4) are shown (a to d). Mock-inoculated plants (with *W*=0) are represented by blue dots. Plants inoculated with the initial PVY variants are represented by green dots, and the slope of the green dotted lines represents their tolerance. Plants inoculated with the final populations are represented by light red or bright red dots when their tolerance (solid lines) is significantly lower or higher, respectively, than the tolerance to the corresponding initial variant. Dotted grey lines represent plant tolerance to final populations that are not significantly different from the tolerance of the same pepper line to the corresponding initial variant.

Overall, when PVY evolution affected plant tolerance, it also frequently affected PVY virulence (assessed as a decrease in *Fw*) but rarely the systemic viral load. *ΔTOL*, the difference in pepper tolerance between final PVY populations and their respective initial variants, was significant for 12 of the 56 (21.4%) evolutionary lineages based on LRTs (Table S4). Pepper tolerance was significantly lower (*ΔTOL*<0) for three final populations corresponding to combination K223 (Fig. 3a), two populations corresponding to combination K2397 (Fig. 3d) and two populations corresponding to combination GK2397 (Fig. 3c). Of these seven final PVY populations, one showed a increase in replicative fitness (population P6 of combination K223), four showed a significant increase in virulence (populations P7 and P8 of combination K2397 and populations P2 and P8 of combination GK2397) and two showed no significant variation from their respective initial variants (data not shown). Conversely, pepper tolerance was significantly higher (*ΔTOL*>0) for five populations of combination GK2334 (Fig. 3b), compared with the initial variant. Of these five final PVY populations, P6 and P8 also showed a significant or marginally significant decrease in virulence, and three populations showed no significant variation in virulence or replicative fitness (data not shown). No significant differences in tolerance were observed for the final PVY populations corresponding to combinations GK223, K253 and K2341, compared with their respective initial variants.

The overall changes in PVY replicative fitness and virulence observed during the EE, and the differences between plant tolerance to final PVY populations and to initial variants, are summarized for each plant-virus combination in Fig. S3.

We further analysed the links between differences in tolerance (*ΔTOL*) and changes in PVY virulence (*ΔVIR*) or replicative fitness (*ΔW*) using correlation analyses. *ΔTOL* was significantly correlated with *ΔVIR* (Pearson’s *r*=−0.60 or −0.70, using independent or joint estimation of tolerance to final PVY populations and to the corresponding initial variant; *P*<1.0e−06) but not with *ΔW* (Pearson’s *r*=0.05 or 0.06, depending on tolerance estimation method, *P*>0.66).

### Infection dynamics in inoculated leaves partly explain changes in replicative fitness and virulence over the course of PVY EE

To interpret changes in PVY properties (*ΔW*, *ΔVIR* or *ΔTOL*) during EE, we measured several traits linked to resistance of pepper lines at the PVY inoculation stage (*N_e_,* the number of primary infection loci) or during colonization of inoculated leaves (*µ*, the delay to leaf colonization; *k*, the final capacity of colonization; and *s*, the speed of colonization). To interpret the final state of PVY populations (*W_f_*, *VIR_f_* or *TOL_f_*), we also used the initial characteristics of the plant–virus combinations (*W_i_*, *VIR_i_* or *TOL_i_*) as explanatory variables. It should be noted that *W_i_* can also be seen as a measure of the susceptibility, as opposed to resistance, of the pepper lines to systemic PVY infection.

**Fig. 4.**
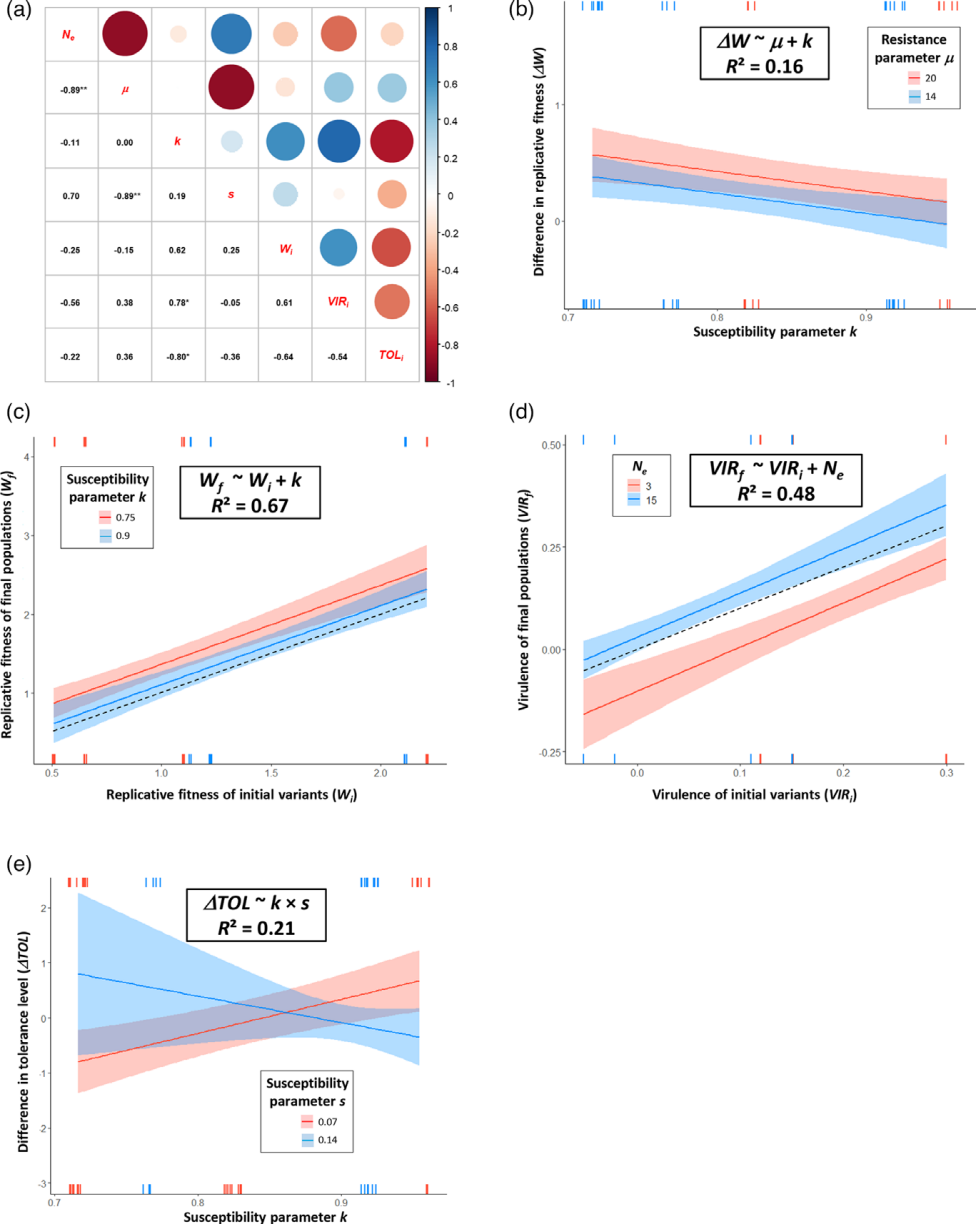
Response of variables related to final properties (*W_f_*, *VIR_f_*), or differences between final and initial properties (*ΔW*, *ΔTOL*), of the plant–virus combinations to variables related to the initial properties of these combinations. (a) Spearman correlation coefficients between explanatory variables *N_e_*, *µ*, *k*, *s*, *W_i_*, *VIR_i_* and *TOL_i_*. Red and blue circles correspond to negative and positive correlations, respectively, and the larger the size of the circle, the stronger the correlation. *P<0.05; **P<0.01. (b to e) Mean responses and confidence bands (in blue and red for lower and higher resistance trait values, respectively) corresponding to generalized linear models 1, 3, 8 and 11, respectively (Tables 1 and S5). Rug plots show the distribution of observed data points along the *x*-axes, with observations with positive residuals at the top and observations with negative residuals at the bottom. The dotted lines correspond to the *W_f_*=*W_i_* (**c**) and *VIR_f_*=*VIR_i_* (**d**) lines. *N_e_* corresponds to the number of infection foci in inoculated leaves, and *µ*, *k* and *s* correspond to the three parameters of the logistic curve representing the colonization of inoculated leaves by PVY (the *y*-value of the asymptote, the *x*-value at the inflection point and the slope at the inflection point after adjustment by *k*, respectively).

We observed strong and significant correlations between explanatory variables, notably between *N_e_* and *µ*, *µ* and *s*, *k* and *VIR_i_* and *k* and *TOL_i_* (Spearman’s correlation coefficient |*ρ*| ≥0.78, Fig. 4a). The strong negative correlation between *N_e_* and *µ* (*ρ*=−0.89) was expected: the lower the number of primary infection loci (*N_e_*), the longer the time to leaf colonization (*µ*). Of the three parameters characterizing the colonization of inoculated leaves, *µ* and *s* were strongly and negatively correlated (*ρ*=−0.89), and *k* was not correlated with the previous ones (|*ρ*| <0.19) and, therefore, represents another type of plant resistance mechanism to PVY. *W_i_* was weakly correlated with *N_e_*, *µ* and *s* (|*ρ*| ≤0.25), indicating that early stages of infection (primary infection foci or early stages of leaf colonization) cannot predict the later stages corresponding to systemic infection.

Because of these correlations, we expected multicollinearity problems when GLMs included several of these variables. Indeed, no GLM with more than three explanatory variables satisfied multicollinearity based on VIF analysis. Table S5 presents the GLMs obtained after a model selection step and with a VIF <6.0. In the following, we will present the subset of these GLMs that satisfied (or almost satisfied) the assumption of normality of residuals based on the Shapiro–Wilk test and that had the lowest cAIC and/or highest *R*² (Table 1 and Fig. 4b–e).

**Table 1.** Parameters of the GLM selected for variables related to PVY evolution or to host plant tolerance GLM properties and variable names are detailed in Table S5. *P<0.05; **P<0.01; ***P<0.001.

Explanatory variable	Estimate	Std. error	*t*-value	***P*-value**
**GLM 1: *ΔW* ~ *µ*+*k***				
Intercept	1.17135	0.51936	2.255	0.02826*
*µ*	0.03148	0.01559	2.020	0.04847*
*k*	−1.71796	0.61645	−2.787	0.00737**
**GLM 3: *W_f_*~*W_i_*+*k***				
Intercept	1.5378	0.5360	2.869	0.0059**
*W_i_*	1.0779	0.1074	10.035	7.44e−14***
*k*	−1.6726	0.7015	−2.384	0.0207*
**GLM 8: *VIR_f_*~*VIR_i_*+*N_e_***				
Intercept	−0.134536	0.043509	−3.092	0.003**
*VIR_i_*	1.076592	0.153846	6.998	4.5e−09***
*N_e_*	0.010989	0.002613	4.206	1.0e−04***
**GLM 11: *ΔTOL*~*k*+*s*+*k*×** ***s***				
Intercept	−14.693	5.378	−2.732	0.00858**
*k*	17.171	5.933	2.894	0.00554**
*s*	135.272	61.830	2.188	0.03320*
*k*×*s*	−157.020	67.713	−2.319	0.02437*

To explain *ΔW*, the selected GLM (*ΔW* ~ *µ+k*) indicates that gains in replicative fitness (*ΔW*>0) during the EE increase with plant resistance to colonization of inoculated leaves (higher values of *µ* or lower values of *k*) and that the effects of *µ* and *k* are cumulative, with no significant interaction between them (Fig. 4b). To explain *W_f_*, the selected model (*W_f_* ~*W_i_+* k) indicates that the replicative fitness of final populations (*W_f_*) increases with plant resistance at the inoculated leaf level (lower *k* values) and increases with replicative fitness of initial PVY variant (*W_i_*), an indicator of the level of plant susceptibility at the systemic level (Fig. 4c). The effects of *W_i_* and *k* are cumulative, with no significant interaction between them.

While none of the models selected to explain *ΔVIR* passed (or almost passed) the Shapiro–Wilk test for normality of residuals (data not shown), the model selected to explain *VIR_f_* (*VIR_f_* ~*VIR_i_+ N_e_*) indicates that the virulence of final PVY populations decreases with plant resistance to inoculation (lower *N_e_* values) and increases with initial PVY virulence (*VIR_i_*) (Fig. 4d). Again, there is no significant interaction between *VIR_i_* and *N_e_*.

While none of the models selected to explain *TOL_f_* passed (or almost passed) the Shapiro–Wilk test for normality of residuals (data not shown), the model selected to explain *ΔTOL* involves an antagonistic interaction between *k* and *s* (Fig. 4e). This model indicates that *ΔTOL* decreases with higher *k* and *s* values (the two variables contributing to greater susceptibility to PVY in inoculated leaves) or with lower *k* and *s* values (the two variables contributing to greater resistance to PVY in inoculated leaves). On the opposite, *ΔTOL* increases with higher *k* and lower *s* values or vice versa.

The models selected to explain *W_f_* and *VIR_f_* had fairly high *R*² values (>0.48), but the *R*² values of the models selected to explain *ΔW* and *ΔTOL* were lower (<0.22) (Table S5).

**Fig. 5.**
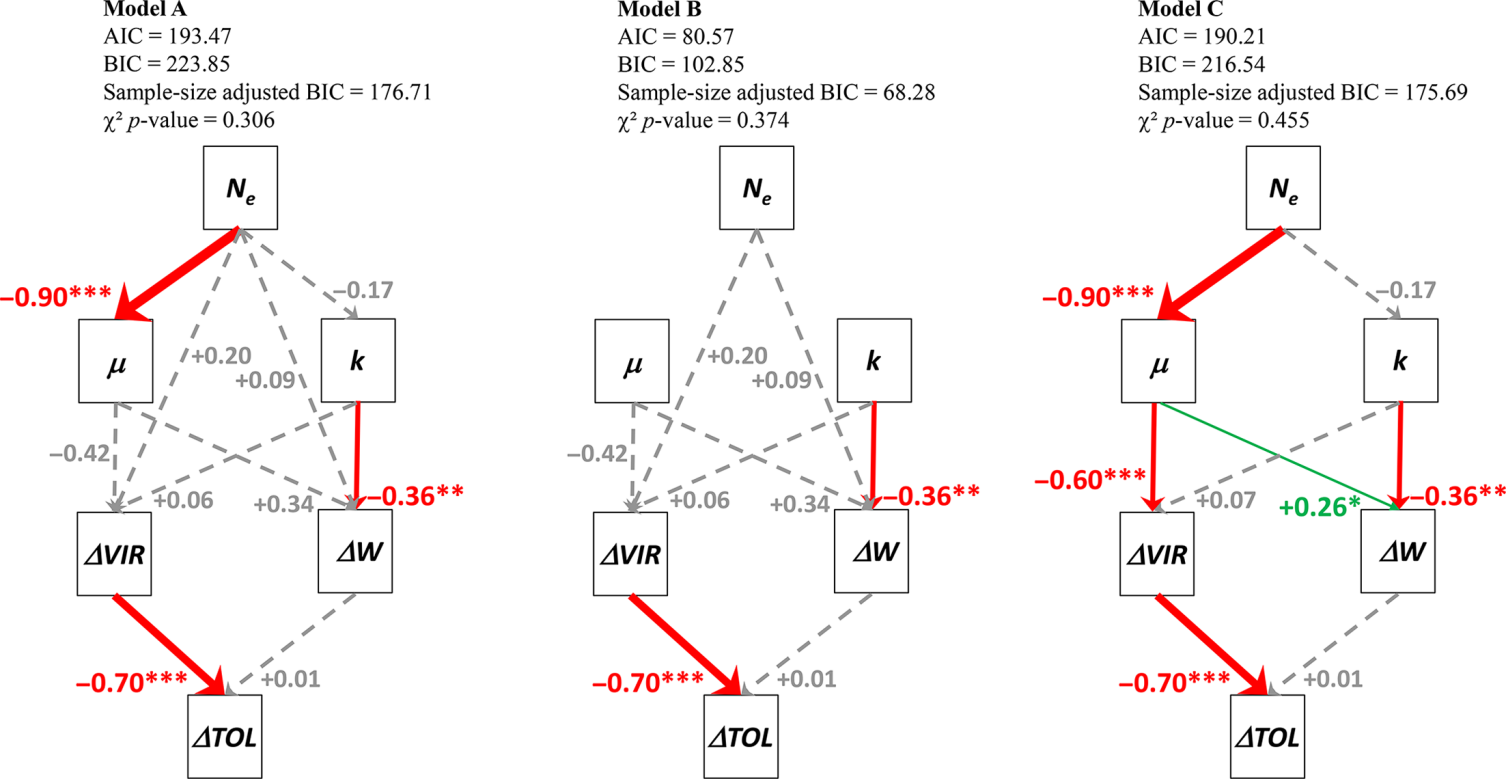
Path diagrams depicting the role of predictor traits associated with pepper resistance (*N_e_*, *μ* and *k*) leading to PVY experimental evolution (response variables *ΔW*, *ΔVIR* and *ΔTOL*). Numbers next to each arrow correspond to standardized regression weights. Red and green solid arrows correspond to significant negative and positive links (*P* < 0.05*, *P* < 0.01**, *P* < 0.001***), respectively, with arrow thickness corresponding to their magnitude, and grey dashed arrows correspond to non-significant links (*P*>0.10). *N_e_* was modelled to have both direct and indirect effects (through accelerating the PVY colonization of inoculated leaves, i.e. reducing *μ*, or increasing their carrying capacity *k*) on *ΔW* and *ΔVIR* (model A) or to have only direct (model B) or indirect (model C) effects. The values of AIC and BIC are shown numerically; the smallest values in model B indicate this is the best-fitting model. Non-significant (*P*>0.05) χ² tests, among other indicators, show good model fit.

Path analyses allowed us to test more complex and realistic models than GLMs to postulate causal relationships between variables. All models incorporating the variables *k*, *μ* and *s* showed a poor fit (data not shown), perhaps because *μ* and *s* are highly correlated (Fig. 4a). Consequently, we excluded the *s* variable from further analyses. The most complex models, including the response variables *ΔTOL*, *ΔW* and *ΔVIR,* gave the same overall results as the combination of the three simple models, including only one of these variables, and showed good model fit, whatever the indicator (chi-square test, comparative fit index, Tucker–Lewis index, root mean square error of approximation or standardized root mean square residual), which is why we will only present these complex models (Fig. 5). Model B, where *N_e_* has only direct effects on *ΔW* and *ΔVIR*, had the lowest AIC and Bayesian information criteria (BIC) and was therefore selected. According to this model, *ΔVIR* has a significant negative effect on *ΔTOL*, and *k* has a significant negative effect on *ΔW. N_e_* has no significant effects on *ΔW* or *ΔVIR.*

## Discussion

### Using experimental evolution to estimate the durability potential of quantitative resistance to PVY in pepper

Despite interest in quantitative resistance controlled by QTLs, data on their durability potential are disparate [9]. For viruses, the complete adaptation of PVY to quantitative resistance in pepper was observed after a few months of EE in the laboratory [10], while the complete breakdown of quantitative resistance to lettuce mosaic virus was only observed after 20 years of commercial use in lettuce cultivars [11]. A few studies conducted on different types of parasites (virus, fungus or nematode) have also highlighted the protective effect of resistance QTLs on the durability potential of a major-effect resistance gene [6,7, 35,38]. Data on the durability potential of plant tolerance to parasites are particularly scarce [39]. In theory, since tolerance does not affect the load and fitness of parasites, but only the damage they cause to plants, it should not impose strong selection on parasite populations and should have a high durability potential. However, parasite mutations that reduce the tolerance level of plants can be fixed in populations by indirect selection if these mutations are associated, by pleiotropy or linkage, with other traits linked to parasite fitness, such as transmission. Indeed, a weakening of the tolerance conferred by the *Zym* gene in commercial zucchini cultivars has been observed following the evolution of ZYMV [20].

EE is an interesting method for better understanding the durability potential of quantitative resistance and tolerance by comparing different plant accessions under the same environmental conditions, with the same inoculum and under the same protocol of successive infection cycles. This method has been successfully used to compare the durability potential of several pepper lines carrying major-effect PVY resistance genes [26,40, 41] or combinations of major genes and QTLs [25].

Montarry *et al.* [10] previously showed that a PVY clone (SON41p) was able to adapt after eight successive infection cycles of 1 month each in the HD223 pepper line. Under the same experimental conditions, no adaptation was visible in the susceptible pepper line ‘Yolo Wonder’ or when the virus was transmitted alternately in HD223 and ‘Yolo Wonder’. In the present study, we extend EE to a more diverse set of quantitatively resistant and tolerant lines, and by analysing the impact of virus evolution on plant tolerance.

The evolutionary trajectories of PVY during EE differed significantly between DH lines (Fig. 2). Indeed, we did not observe any significant changes in virulence (*ΔVIR*) or replicative fitness (*rW*) between the initial PVY variants and the 8 final populations that evolved in HD253 or HD2341 (Fig. 2). On the contrary, we observed a significant overall increase in viral replicative fitness, particularly for 5 of the 16 final populations, in HD223, the most resistant line at systemic level, and a significant overall decrease in virulence, particularly for 2 of the 8 final populations, in HD2334, the most resistant line at inoculation stage. We also observed a significant (or marginally significant) increase in virulence for 5 of 16 populations evolved in HD2397. In contrast, we noted little difference between the two initial PVY variants K and GK when they evolved on the same DH line, probably due to the similarity of their genomes and replicative fitness *W_i_* (Table S1).

This study confirms the ability of PVY to adapt to the resistance of the pepper line HD223 during a few months of EE, as already described by Montarry *et al.* [10]. However, in the present study, the increase in PVY replicative fitness was smaller (two- to threefold vs. ~12-fold in Montarry *et al.* [10]) and affected fewer evolutionary lineages (5/16 vs. 10/10) (Fig. 2a, c). These differences could be due to differences in the initial PVY variants (SON41p in Montarry *et al.* [10] and variants K and GK of SON41p in the present study) or in the environmental conditions (climatic chamber with controlled climate vs. greenhouse). In both studies, we did not observe significant changes in PVY virulence in HD223, which could be due to the high level of tolerance of this pepper line (Fig. 3, Table S3) [10].

The fact that no significant changes in PVY replicative fitness or virulence were observed for PVY populations evolved in HD253 or HD2341 could be due to the absence of resistance alleles in these pepper lines and to moderate or high susceptibility and tolerance to systemic PVY infection (Tables S1 and S3).

In HD2334, PVY virulence decreased overall during EE (Fig. 2b), particularly in the two final populations P6 and P8 (Fig. 2c). This result is not affected by the fact that several final populations shared the same ancestry until the end of the first infection cycle. Indeed, P6 and P8 have evolved independently since the beginning of the EE (Fig. 1). In contrast, in HD2397, PVY virulence increased overall, and more specifically in five final populations (Fig. 2b, c). Surprisingly, for HD2334 and HD2397, where PVY virulence changed significantly, we observed no significant changes in replicative fitness.

These results also suggest that changes in virulence and replicative fitness during PVY evolution are caused by different mutations and may involve different viral proteins. The fact that the two variables *ΔW* and *ΔVIR* are not significantly correlated also supports this hypothesis.

### Impact of PVY evolution on pepper tolerance levels

Interestingly, the level of plant tolerance can differ significantly depending on whether it has been estimated with an initial PVY variant or a final population, giving some indication of tolerance durability potential. Data on the durability potential of plant tolerance are very scarce, and it is a debated topic [18]. Indeed, plant tolerance is often estimated qualitatively: a tolerant plant genotype shows no damage and/or no loss of fitness or production despite a high pathogen load. Consequently, plant tolerance does not affect the pathogen’s ability to replicate in the plant, and there is no obvious advantage for the pathogen in altering the plant’s tolerance level. Quantitative estimates of tolerance, such as the reaction norm represented by the slope of the relationship between a measure of host health (here *Fw*) and pathogen intra-host load (*W*) [42], are, therefore, needed to better estimate the links between host tolerance and pathogen fitness, as well as the impact of pathogen evolution on host tolerance. This latter point will be discussed below. We proposed an original method for testing the significance of the difference in plant tolerance before and after PVY evolution, by comparing two linear regression models representing range tolerance, one model considering that the slope of the regression was different between the final populations and the initial variant and a null model considering that the slope was similar. While HD223 is highly tolerant and HD2397 rather intolerant to PVY infection (Table S1), we observed downward trends in the tolerance of (i) HD223 to PVY populations evolved from initial variant K (3 of 8 populations) and (ii) HD2397 to PVY populations evolved from either initial variant K or GK (4 of 16 populations) (Fig. 3a, c, d). Conversely, while HD2334 has a low tolerance towards initial variant GK (Table S3), we observed a consistently higher tolerance to the evolved PVY populations (5 of 8 populations, Fig. 3b). Significant differences in plant tolerance to evolved vs. initial PVY were associated with changes in PVY virulence in six cases, rarely with changes in replicative fitness (one case) and were not associated with any significant change in virulence or replicative fitness in the remaining five cases. Overall, these results suggest that the evolution of PVY with regard to pepper tolerance is rather independent of initial levels of PVY virulence or replicative fitness. Accordingly, model selection discarded the variables *VIR_i_* or *W_i_* to explain the variable *△TOL* (Tables 1 and S5).

Wollein Waldetoft *et al.* [43] introduced the term ‘benevolence’ (or ‘malevolence’) as the microbial equivalent of host tolerance. Benevolence (and malevolence) thus represents the positive (respectively negative) effect a microbe exerts on a host’s fitness or health relative to its load. Virulence is, therefore, the product of a microbe’s malevolence and intra-host load. According to this view, host tolerance targets parasite benevolence, rather than intra-host load (or replicative fitness), and could be a means of preventing parasite counter-adaptation, such as the evolution of antimicrobial resistance, as it leaves the microbe’s density, a key component of its Darwinian fitness, unchanged [43]. The concepts of malevolence and benevolence apply to plant viruses and viruses in general. Viruses can evolve along a ‘mutualist—parasite continuum’ [44]. While agronomists have focused on pathogenic plant viruses, ecologists have demonstrated the positive effects of plant viruses on the health or fitness of their hosts [45]. Here, taking into account the intra-host viral load, we show that viruses also evolve along a benevolence—malevolence continuum.

Indeed, we show here that PVY benevolence can evolve rapidly and in different ways depending on its host (Fig. 3, Table S3). In HD223, an overcompensating pepper line, the benevolence of PVY variant K decreased during the EE. In the intolerant line HD2334, the malevolence of PVY variant GK decreased during the EE. Finally, in the intolerant line HD2397, the malevolence of PVY variants K and GK increased or remained stable, depending on the final population, during the EE. Little change in PVY malevolence or benevolence was observed in the other pepper-PVY combinations.

### Insights into mechanisms determining PVY evolutionary trajectories

In order to study the mechanisms that determined PVY evolutionary trajectories, we have used several parameters linked to the plant’s level of resistance acting at the different stages of viral infection: resistance to inoculation estimated by *N_e_*; resistance to leaf colonization estimated by parameters *µ*, *k* and *s* representing PVY growth curve; and resistance to systemic infection, inversely related to *W_i_*. GLM analyses revealed that gains in replicative fitness (*ΔW*) and final replicative fitness (*W_f_*) were higher in pepper lines with the highest levels of resistance to leaf colonization (parameters *k* and/or *µ*) (Fig. 4).

We cannot rule out the existence of potential biases in the estimation of the parameters *µ*, *k* and *s*, relative to the expected values for wild-type PVY, which could be related to the presence of the GFP transgene and its differential effects on PVY infection in the different pepper lines. However, analyses showed that *µ*, *k* and *s* were relevant for explaining PVY evolution trajectories, suggesting that these potential biases are small or inexistent.

Tamisier *et al.* [25] carried out an EE study with variants of SON41p, including variant K used in the present study, on pepper lines possessing the major-effect resistance gene *pvr2^3^* associated with different genetic backgrounds. Evolutionary trajectories were well explained by host-imposed genetic drift during systemic infection, a parameter that we did not estimate in the present study, and the initial replicative fitness, *W_i_*. Virus adaptation (high *ΔW*) mainly occurred and was more important when both genetic drift and *W_i_* were low. We have confirmed here that virus adaptation is greater in more resistant plants (low *W_i_*), although using other resistance parameters related to leaf colonization. According to EE studies, the probability, speed and/or intensity of adaptation are higher for parasite strains with a low replicative fitness (estimated here with systemic viral load *W* and corresponding to highly resistant hosts) than for strains with high fitness [46]. Indeed, strains with low fitness can fix beneficial mutations with greater effects and at a higher rate, compared with high-fitness strains [47,48]. In addition, the ratio between the number of beneficial and deleterious mutations may be higher in strains with low replicative fitness [49]. Compared with Tamisier *et al*. [25], we observed no link between *ΔW* (or *W_f_*) and genetic drift (*N_e_*). This may be due to the fact that *µ*, representing the delay in leaf colonization by PVY, and *N_e_* are highly correlated (*r*=−0.89, Fig. 4a) and *µ* (but not *N_e_*) has been retained in the GLM to explain *ΔW* (Table S5). The virulence of the final PVY populations was lower in pepper lines with the narrowest bottlenecks during PVY inoculation (low *N_e_*) and in those where the initial PVY variants had low virulence. This raises the question of what selective forces have driven PVY virulence evolution. One possibility is that the use of mechanical inoculation during EE relaxed the constraints imposed by the natural mode of virus transmission (i.e*.* by aphid vectors), which may have affected PVY virulence without altering replicative fitness. PVY mutations with pleiotropic effects, simultaneously affecting virulence and aphid transmission (e.g*.* in the multifunctional HcPro protein) [50] could be responsible for these PVY evolutionary trajectories. In addition, the opposite trend in virulence evolution in HD2334 and HD2397 could be due to the contrasting tolerance levels of these lines (Table S1).

The evolution of parasite virulence is the subject of debate, as virulence, represented by the damage caused to the host, has generally no obvious advantage for parasites, except necrotrophic or hemibiotrophic plant bacteria, fungi or oomycetes. Most models assume that virulence is an inevitable consequence of parasite intra-host multiplication, leading to trade-offs between intra-host multiplication and inter-host transmission [51]. However, the central assumption of these models, namely a positive correlation between intra-host multiplication and virulence, is poorly confirmed experimentally for plant–parasite systems [52]. This could be due to tolerance mechanisms determined by host genotypes. In our case, narrow bottlenecks could have been responsible for stronger genetic drift and subsequent Muller’s ratchet process [53,54], where deleterious mutations could have accumulated in the PVY genome, without being purged by recombination, resulting in reduced virulence. However, this would also imply a reduction in PVY fitness, which is not supported by the lack of significant correlation between *ΔW* and *ΔVIR* for the evolved PVY populations. Another hypothesis is that genetic drift associated with low *N_e_* values may have accelerated the fixation of PVY mutations with pleiotropic effects on both virulence and fitness traits other than replicative fitness, such as inter-host transmission.

Identification of the PVY mutations responsible for changes in *W* or *VIR* during the EE will be necessary to decipher the mechanisms involved in PVY evolutionary trajectories and the links between changes in virulence and different fitness traits.

## Conclusion

This study provides information on the potential durability of quantitative resistance and tolerance to PVY in pepper. It shows that PVY adaptation (measured by an increase in systemic viral load) can occur rapidly in a pepper line (HD223) with high resistance to inoculated leaf colonization and that this adaptation has no detrimental effect on the plant in terms of biomass. An increase in PVY virulence was observed in one of the least tolerant pepper lines (HD2397), which was not associated with a significant change in replicative fitness and was mainly determined by susceptibility to PVY inoculation (a high number of primary infection foci). Finally, our results demonstrate that the benevolence (or malevolence) of PVY changed significantly during the EE in several pepper lines, thereby altering pepper tolerance and calling into question the durability potential of plant tolerance to viruses.

## Supplementary material

10.1099/jgv.0.002208 Uncited Supplementary Material 1.

10.1099/jgv.0.002208 Uncited Supplementary Material 2.

## Abbreviations

AIC: Akaike’s information criterion
BIC: Bayesian information criterion
cAIC: corrected Akaike information criterion
DAS-ELISA: double-antibody sandwich ELISA
DH: doubled-haploid
dpi: days post-inoculation
EE: experimental evolution
GFP: green fluorescent protein
GLM: generalized linear model
LRT: likelihood ratio test
PVY: potato virus Y
QTL: quantitative trait locus
VIF: variance inflation factor
ZYMV: zucchini yellow mosaic virus

## References

[1] Foxe MJ (1992). Breeding for viral resistance: conventional methods. Neth J Plant Pathol.

[2] Gómez P, Rodríguez-Hernández AM, Moury B, Aranda MA (2009). Genetic resistance for the sustainable control of plant virus diseases: breeding, mechanisms and durability. Eur J Plant Pathol.

[3] Lecoq H, Moury B, Desbiez C, Palloix A, Pitrat M (2004). Durable virus resistance in plants through conventional approaches: a challenge. Virus Res.

[4] Chain F, Riault G, Jacquot E, Trottet M (2006). Field trial of serially passaged isolates of BYDV‐PAV overcoming resistance derived from *Thinopyrum intermedium* in wheat. Plant Breed.

[5] Michel V, Julio E, Candresse T, Cotucheau J, Decorps C (2019). A complex *eIF4E locus* impacts the durability of *va* resistance to *Potato virus Y* in tobacco. Mol Plant Pathol.

[6] Palloix A, Ayme V, Moury B (2009). Durability of plant major resistance genes to pathogens depends on the genetic background, experimental evidence and consequences for breeding strategies. New Phytol.

[7] Quenouille J, Saint-Felix L, Moury B, Palloix A (2016). Diversity of genetic backgrounds modulating the durability of a major resistance gene. Analysis of a core collection of pepper landraces resistant to *Potato virus Y*. Mol Plant Pathol.

[8] Rousseau E, Bonneault M, Fabre F, Moury B, Mailleret L (2019). Virus epidemics, plant-controlled population bottlenecks and the durability of plant resistance. *Philos Trans R Soc Lond B Biol Sci*.

[9] Cowger C, Brown JKM (2019). Durability of quantitative resistance in crops: greater than we know?. Annu Rev Phytopathol.

[10] Montarry J, Cartier E, Jacquemond M, Palloix A, Moury B (2012). Virus adaptation to quantitative plant resistance: erosion or breakdown?. J Evol Biol.

[11] Pink DAC, Lot H, Johnson R (1992). Novel pathotypes of lettuce mosaic virus — breakdown of a durable resistance?. Euphytica.

[12] Pagán I, García-Arenal F (2020). Tolerance of plants to pathogens: a unifying view. Annu Rev Phytopathol.

[13] Robinson RW, Provvidenti R (1997). Differential response of *Cucurbita pepo* cultivars to strains of *Zucchini yellow mosaic virus*. Cucurbit Genet Coop Rep.

[14] Michel V, Julio E, Candresse T, Cotucheau J, Decorps C (2018). NtTPN1: a RPP8-like R gene required for *Potato virus Y*-induced veinal necrosis in tobacco. Plant J.

[15] Shukla A, Pagán I, Crevillén P, Alonso-Blanco C, García-Arenal F (2022). A role of flowering genes in the tolerance of *Arabidopsis thaliana* to cucumber mosaic virus. Mol Plant Pathol.

[16] Tamisier L, Szadkowski M, Girardot G, Djian-Caporalino C, Palloix A (2022). Concurrent evolution of resistance and tolerance to *Potato virus Y* in *Capsicum annuum* revealed by genome-wide association. Mol Plant Pathol.

[17] Zinger A, Lapidot M, Harel A, Doron-Faigenboim A, Gelbart D (2021). Identification and mapping of tomato genome loci controlling tolerance and resistance to *Tomato brown rugose fruit virus*. Plants.

[18] Pagán I, García-Arenal F (2018). Tolerance to plant pathogens: theory and experimental evidence. Int J Mol Sci.

[19] Råberg L, Sim D, Read AF (2007). Disentangling genetic variation for resistance and tolerance to infectious diseases in animals. Science.

[20] Desbiez C, Gal-On A, Girard M, Wipf-Scheibel C, Lecoq H (2003). Increase in *Zucchini yellow mosaic virus* symptom severity in tolerant Zucchini cultivars is related to a point mutation in P3 protein and is associated with a loss of relative fitness on susceptible plants. Phytopathology.

[21] Quenouille J, Paulhiac E, Moury B, Palloix A (2014). Quantitative trait loci from the host genetic background modulate the durability of a resistance gene: a rational basis for sustainable resistance breeding in plants. Heredity.

[22] Salinier J, Lefebvre V, Besombes D, Burck H, Causse M (2022). The INRAE centre for vegetable germplasm: geographically and phenotypically diverse collections and their use in genetics and plant breeding. Plants.

[23] Moury B, Morel C, Johansen E, Guilbaud L, Souche S (2004). Mutations in *Potato virus Y* genome-linked protein determine virulence toward recessive resistances in *Capsicum annuum* and *Lycopersicon hirsutum*. Mol Plant Microbe Interact.

[24] Rousseau E, Moury B, Mailleret L, Senoussi R, Palloix A (2017). Estimating virus effective population size and selection without neutral markers. PLoS Pathog.

[25] Tamisier L, Fabre F, Szadkowski M, Chateau L, Nemouchi G (2024). Within-plant genetic drift to control virus adaptation to host resistance genes. PLoS Pathog.

[26] Ayme V, Souche S, Caranta C, Jacquemond M, Chadoeuf J (2006). Different mutations in the genome-linked protein VPg of *Potato virus Y* confer virulence on the pvr2(3) resistance in pepper. Mol Plant Microbe Interact.

[27] Elena SF (2017). Local adaptation of plant viruses: lessons from experimental evolution. Mol Ecol.

[28] Wargo AR, Kurath G (2012). Viral fitness: definitions, measurement, and current insights. Curr Opin Virol.

[29] Moghal SM, Francki RIB (1981). Towards a system for the identification and classification of potyviruses. II. Virus particle length, symptomatology, and cytopathology of six distinct viruses. Virology.

[30] Tamisier L, Rousseau E, Barraillé S, Nemouchi G, Szadkowski M (2017). Quantitative trait loci in pepper control the effective population size of two RNA viruses at inoculation. J Gen Virol.

[31] Zwart MP, Daròs J-A, Elena SF (2011). One is enough: in vivo effective population size is dose-dependent for a plant RNA virus. PLoS Pathog.

[32] Jackson DA, Somers KM (1991). The spectre of “spurious” correlations. Oecologia.

[33] Streiner DL (2005). Finding our way: an introduction to path analysis. Can J Psychiatry.

[34] Masini L, Grenville-Briggs LJ, Andreasson E, Råberg L, Lankinen Å (2019). Tolerance and overcompensation to infection by *Phytophthora infestans* in the wild perennial climber *Solanum dulcamara*. Ecol Evol.

[35] Brun H, Chèvre A-M, Fitt BDL, Powers S, Besnard A-L (2010). Quantitative resistance increases the durability of qualitative resistance to *Leptosphaeria maculans* in *Brassica napus*. New Phytol.

[36] Pilet-Nayel M-L, Moury B, Caffier V, Montarry J, Kerlan M-C (2017). Quantitative resistance to plant pathogens in pyramiding strategies for durable crop protection. Front Plant Sci.

[37] Quenouille J, Montarry J, Palloix A, Moury B (2013). Farther, slower, stronger: how the plant genetic background protects a major resistance gene from breakdown. Mol Plant Pathol.

[38] Rousseau E, Tamisier L, Fabre F, Simon V, Szadkowski M (2018). Impact of genetic drift, selection and accumulation level on virus adaptation to its host plants. Mol Plant Pathol.

[39] Koch KG, Chapman K, Louis J, Heng-Moss T, Sarath G (2016). Plant tolerance: a unique approach to control hemipteran pests. Front Plant Sci.

[40] Janzac B, Montarry J, Palloix A, Navaud O, Moury B (2010). A point mutation in the polymerase of *Potato virus Y* confers virulence toward the Pvr4 resistance of pepper and a high competitiveness cost in susceptible cultivar. Mol Plant Microbe Interact.

[41] Moury B, Michon T, Simon V, Palloix A (2023). A single nonsynonymous substitution in the RNA-dependent RNA polymerase of *Potato virus Y* allows the simultaneous breakdown of two different forms of antiviral resistance in *Capsicum annuum*. Viruses.

[42] Råberg L (2014). How to live with the enemy: understanding tolerance to parasites. PLoS Biol.

[43] Wollein Waldetoft K, Råberg L, Lood R (2020). Proliferation and benevolence-A framework for dissecting the mechanisms of microbial virulence and health promotion. Evol Appl.

[44] Alizon S, Turner PE (2021). Can we eradicate viral pathogens?. J Evol Biol.

[45] Roossinck MJ (2019). Viruses in the phytobiome. Curr Opin Virol.

[46] Navarro R, Ambrós S, Butković A, Carrasco JL, González R (2022). Defects in plant immunity modulate the rates and patterns of RNA virus evolution. Virus Evol.

[47] Barrick JE, Kauth MR, Strelioff CC, Lenski RE (2010). *Escherichia coli rpoB* mutants have increased evolvability in proportion to their fitness defects. Mol Biol Evol.

[48] Moore FB-G, Rozen DE, Lenski RE (2000). Pervasive compensatory adaptation in *Escherichia coli*. Proc R Soc Lond B.

[49] Silander OK, Tenaillon O, Chao L (2007). Understanding the evolutionary fate of finite populations: the dynamics of mutational effects. PLoS Biol.

[50] Valli AA, Gallo A, Rodamilans B, López-Moya JJ, García JA (2018). The HCPro from the *Potyviridae* family: an enviable multitasking helper component that every virus would like to have. Mol Plant Pathol.

[51] Alizon S, Hurford A, Mideo N, Van Baalen M (2009). Virulence evolution and the trade-off hypothesis: history, current state of affairs and the future. J Evol Biol.

[52] Pagán I, Alonso-Blanco C, García-Arenal F (2007). The relationship of within-host multiplication and virulence in a plant-virus system. PLoS One.

[53] de la Iglesia F, Elena SF (2007). Fitness declines in *Tobacco etch virus* upon serial bottleneck transfers. J Virol.

[54] Gordo I, Charlesworth B (2000). The degeneration of asexual haploid populations and the speed of Muller’s ratchet. Genetics.

