# Supplementary material for: The replicative fitness and virulence of potato virus Y evolve differently in pepper lines with different levels of resistance and tolerance
Source: J Gen Virol. 2026 Feb 2;107(2):002208. doi: 10.1099/jgv.0.002208 (PMC12863973; doi:10.1099/jgv.0.002208)
Supplement: Uncited Supplementary Material 2. [file jgv-107-02208-s002.pdf]

**Table S1. Parameters of PVY resistance of the five pepper lines of the present study and of the susceptible reference line Yolo Wonder.**

| Pepper line | Inoculation stage[1] | Colonization of the inoculated leaf[2] | | | Systemic infection[3] | Alleles at QTLs[4] | | |
|---|---|---|---|---|---|---|---|---|
| | $N_e$ | $k$ | $\mu$ (day) | $s$ ($10^{-2}$.day$^{-1}$) | $W_i$ | **QTL6** | **QTLs 8 + 9** | **QTL9** |
| HD223 | 14.438 | 0.716 | 15.4 | 7.9 | 0.509 (GK) or 0.652 (K) | R | 0 | Tol |
| HD253 | 8.500 | 0.768 | 14.7 | 11.3 | 2.213 (K) | (rec) | 0 | Tol |
| HD2334 | 0.125 | 0.954 | 24.0 | 6.9 | 2.115 (GK) | (rec) | S | Tol |
| HD2341 | 4.250 | 0.824 | 17.7 | 9.2 | 1.099 (K) | S | S | Tol |
| HD2397 | 17.867 | 0.920 | 12.4 | 15.4 | 1.131 (GK) or 1.223 (K) | R | 0 | Intol |
| Yolo Wonder | 30.389 | 0.867 | 9.0 | 23.7 | NA[5] | S | S | Tol |

[1] $N_e$ is the size of the bottleneck imposed by the pepper line on the PVY population at the inoculation stage and is estimated by the number of primary infection foci visualized with a GFP-tagged variant of SON41p-115K (variant K) after mechanical inoculation of pepper leaves, as in [30].

[2] $\mu$, $k$ and $s$ are the three parameters of the Verhulst growth model fitted to leaf colonization by the same GFP-tagged PVY variant after mechanical inoculation (see Fig. S1 for details).

[3] $W_i$ is the systemic viral load of PVY variant K or GK estimated in the present study.

[4] The presence of resistance or tolerance alleles at QTLs identified by Quenouille-Lederer [55] and Quenouille et al. [20], named based on the chromosome number of their location, is indicated. R: resistance allele based on systemic viral load measurement; 0: allelic combination with no effect on resistance level (case of epistatic QTLs 8 and 9); Tol: tolerance allele (induction of systemic mosaic upon PVY infection); (rec): the QTL region has been affected by recombination, making it impossible to identify the allele present at this QTL; S: susceptibility allele; Intol: intolerance allele (induction of systemic necrosis upon PVY infection).

[5] NA: not available. Yolo Wonder was not considered for $W_i$ measurement in the present study. $W_i$ of another PVY variant (SON41p) was between two and three times higher in Yolo Wonder than in HD223 [6,10].

## Additional reference

**55.** **Quenouille-Lederer J.** *Bases génétiques et fonctionnelles de la durabilité des résistances polygéniques au virus Y de la pomme de terre (PVY) chez le piment (Capsicum annuum L.)*. PhD thesis, Avignon Université, France, 2013, 214 pp.

**Table S2.** Versions of the R packages used.

| Package | Version |
|---|---|
| abind | 1.4-8 |
| aplpack | 1.3.5 |
| arm | 1.14-4 |
| backports | 1.5.0 |
| base64enc | 0.1-3 |
| bestNormalize | 1.9.1 |
| boot | 1.3-30 |
| cachem | 1.1.0 |
| car | 3.1-2 |
| carData | 3.0-5 |
| checkmate | 2.3.2 |
| cli | 3.6.4 |
| cluster | 2.1.6 |
| coda | 0.19-4.1 |
| colorspace | 2.1-1 |
| corpcor | 1.6.10 |
| corrplot | 0.94 |
| cowplot | 1.1.3 |
| data.table | 1.17.0 |
| DescTools | 0.99.54 |
| devtools | 2.4.5 |
| digest | 0.6.37 |
| dplyr | 1.1.4 |
| dvmisc | 1.1.4 |
| ellipsis | 0.3.2 |
| EnvStats | 2.8.1 |
| evaluate | 1.0.3 |
| FactoMineR | 2.10 |
| fastmap | 1.2.0 |
| fdrtool | 1.2.18 |
| forcats | 1.0.0 |
| foreign | 0.8-86 |
| Formula | 1.2-5 |
| fs | 1.6.6 |
| generics | 0.1.3 |
| ggplot2 | 3.5.2 |
| ggpubr | 0.6.0 |
| glasso | 1.11 |
| glue | 1.8.0 |

| | |
|---|---|
| gridExtra | 2.3 |
| gtable | 0.3.6 |
| gtools | 3.9.5 |
| Hmisc | 5.2-3 |
| htmlTable | 2.4.3 |
| htmltools | 0.5.8.1 |
| htmlwidgets | 1.6.4 |
| httpuv | 1.6.15 |
| igraph | 2.1.4 |
| jpeg | 0.1-11 |
| knitr | 1.46 |
| knitr | 1.50 |
| kutils | 1.73 |
| later | 1.4.2 |
| lattice | 0.22-6 |
| lavaan | 0.6-19 |
| lifecycle | 1.0.4 |
| lisrelToR | 0.3 |
| lme4 | 1.1-35.1 |
| lmtest | 0.9-40 |
| lsmeans | 2.30-0 |
| magrittr | 2.0.3 |
| markdown | 1.12 |
| MASS | 7.3-60.2 |
| Matrix | 1.7-0 |
| memoise | 2.0.1 |
| mi | 1.1 |
| mime | 0.13 |
| miniUI | 0.1.1.1 |
| minqa | 1.2.8 |
| missMDA | 1.19 |
| mnormt | 2.1.1 |
| multcomp | 1.4-25 |
| MuMIn | 1.48.4 |
| munsell | 0.5.1 |
| nlme | 3.1-164 |
| nloptr | 2.2.1 |
| nnet | 7.3-19 |
| OpenMx | 2.21.13 |
| openxlsx | 4.2.8 |
| outliers | 0.15 |

| | |
|---|---|
| patchwork | 1.2.0 |
| pbapply | 1.7-2 |
| pbivnorm | 0.6.0 |
| PerformanceAnalytics | 2.0.4 |
| pillar | 1.10.2 |
| pkgbuild | 1.4.7 |
| pkgconfig | 2.0.3 |
| pkgload | 1.4.0 |
| plotly | 4.10.4 |
| plyr | 1.8.9 |
| PMCMRplus | 1.9.10 |
| png | 0.1-8 |
| profvis | 0.4.0 |
| promises | 1.3.2 |
| pscl | 1.5.9 |
| psych | 2.4.3 |
| purrr | 1.0.4 |
| qgraph | 1.9.8 |
| quadprog | 1.5-8 |
| R6 | 2.6.1 |
| rbibutils | 2.3 |
| Rcpp | 1.0.14 |
| Rdpack | 2.6.4 |
| readxl | 1.4.5 |
| reformulas | 0.4.0 |
| remotes | 2.5.0 |
| reshape2 | 1.4.4 |
| rlang | 1.1.6 |
| rlist | 0.4.6.2 |
| rmarkdown | 2.26 |
| rockchalk | 1.8.157 |
| rpart | 4.1.23 |
| rstudioapi | 0.17.1 |
| scales | 1.3.0 |
| sem | 3.1-16 |
| semPlot | 1.1.6 |
| sessioninfo | 1.2.3 |
| shiny | 1.10.0 |
| stringi | 1.8.7 |
| stringr | 1.5.1 |
| tibble | 3.2.1 |

| | |
|---|---|
| tidyr | 1.3.1 |
| tidyselect | 1.2.1 |
| urlchecker | 1.0.1 |
| usethis | 3.1.0 |
| vctrs | 0.6.5 |
| visreg | 2.7.0 |
| xfun | 0.52 |
| XML | 3.99-0.18 |
| xtable | 1.8-4 |
| zip | 2.3.2 |

**Table S3.** Estimation of the range tolerance of pepper DH lines to PVY variants. The tolerance level of pepper DH lines to each final PVY population and initial variant was estimated as the slope of the linear regression between *W* and *Fw*, including or excluding the mock-inoculated plants (for which $W = 0$). Overall, linear regression was more appropriate to represent the relationship between *W* and *Fw* when mock-inoculated plants were included, providing similar or higher |r| values than regressions obtained with PVY-infected plants alone. |r| values were also more frequently significant when mock-inoculated plants were included. The only exception was combination K2341, which showed a significant correlation with PVY-infected plants alone but not when mock-inoculated plants were also included.

| | **Tolerance estimation** | | | | | | **Comparison of tolerance methods** | |
|---|---|---|---|---|---|---|---|---|
| | **With mock-inoc.** | | | **Without mock-inoc.** | | | | |
| **Combination** | $TOL_i$[1] | **r**[2] | ***p***[3] | $TOL_i$[1] | **r**[2] | ***p***[3] | **r'**[4] | ***p'***[5] |
| GK223 | -0.14 | -0.026 | 0.902 | -0.79 | -0.143 | 0.598 | 0.547 | 0.128 |
| K223 | **1.40** | **0.394** | **0.042** | **1.66** | **0.491** | **0.039** | **0.915** | **5.4e-04** |
| K253 | **-0.64** | **-0.450** | **0.047** | 0.09 | 0.044 | 0.905 | **0.681** | **0.043** |
| GK2334 | **-0.97** | **-0.526** | **0.007** | -0.43 | -0.219 | 0.433 | 0.611 | 0.080 |
| K2341 | 0.13 | 0.042 | 0.859 | **2.62** | **0.663** | **0.037** | 0.587 | 0.097 |
| GK2397 | **-0.80** | **-0.454** | **0.012** | **-0.42** | -0.277 | 0.238 | **0.774** | **0.014** |
| K2397 | **-1.00** | **-0.388** | **0.038** | -0.53 | -0.182 | 0.456 | 0.361 | 0.340 |
| All | | | | | | | **0.399** | **0.001** |

[1] Slope of regression between plant fresh weight *Fw* and systemic viral load $W_i$ (initial PVY variants only).

[2] r : Pearson's correlation coefficient between plant fresh weight *Fw* and systemic viral load $W_i$ (initial PVY variants only).

[3] p : Significance of r correlation coefficients.

[4] r' : Pearson's correlation coefficient between tolerance estimates ($TOL_i$ and $TOL_f$ together) obtained with the two methods (with or without mock-inoculated plants).

[5] p' : Significance of correlation coefficients r'.

Values corresponding to significant correlations ($p < 0.05$) are in bold.

**Table S4.** Likelihood ratio tests (LRTs) of two linear models of range tolerance of pepper DH lines to PVY variants, differentiating or not the PVY initial variants and final populations.

| PVY–pepper combination | Final PVY population | LRT (p-value[2]) | Sign of $\Delta TOL$[1] |
|---|---|---|---|
| GK223 | P1 | 0.369 | NS |
| | P2 | 0.773 | NS |
| | P3 | 0.909 | NS |
| | P4 | 0.905 | NS |
| | P5 | 0.907 | NS |
| | P6 | 0.364 | NS |
| | P7 | 0.395 | NS |
| | P8 | 0.428 | NS |
| K223 | P1 | 0.114 | NS |
| | P2 | 0.191 | NS |
| | P3 | 0.018*[2] | Negative |
| | P4 | 0.483 | NS |
| | P5 | 0.004** | Negative |
| | P6 | 0.025* | Negative |
| | P7 | 0.251 | NS |
| | P8 | 0.576 | NS |
| K253 | P1 | 0.108 | NS |
| | P2 | 0.835 | NS |
| | P3 | 0.322 | NS |
| | P4 | 0.800 | NS |
| | P5 | 0.698 | NS |
| | P6 | 0.860 | NS |
| | P7 | 0.360 | NS |
| | P8 | 0.774 | NS |
| GK2334 | P1 | 0.403 | NS |
| | P2 | 0.003** | Positive |
| | P3 | 0.047* | Positive |
| | P4 | 0.077 | NS |
| | P5 | 0.195 | NS |
| | P6 | 0.003** | Positive |
| | P7 | 0.009** | Positive |
| | P8 | 0.005** | Positive |
| K2341 | P1 | 0.876 | NS |
| | P2 | 0.809 | NS |
| | P3 | 0.859 | NS |
| | P4 | 0.939 | NS |
| | P5 | 0.360 | NS |
| | P6 | 0.551 | NS |
| | P7 | 0.992 | NS |
| | P8 | 0.787 | NS |
| GK2397 | P1 | 0.723 | NS |
| | P2 | 0.002** | Negative |
| | P3 | 0.151 | NS |
| | P4 | 0.787 | NS |
| | P5 | 0.606 | NS |
| | P6 | 0.397 | NS |
| | P7 | 0.797 | NS |
| | P8 | 4.e-4*** | Negative |

| | | | |
|---|---|---|---|
| K2397 | P1 | 0.059 | NS |
| | P2 | 0.105 | NS |
| | P3 | 0.580 | NS |
| | P4 | 0.580 | NS |
| | P5 | 0.075 | NS |
| | P6 | 0.274 | NS |
| | P7 | 0.002** | Negative |
| | P8 | 0.001*** | Negative |

[1] The LRT statistics corresponds to -2 × log (*L*(null model) / *L*(full model)), where *L*(null model) and *L*(full model) are the likelihoods of the null and full models (see the Methods section for details), respectively, and was compared with a Chi-squared with one degree of freedom to obtained the p-values. *, **, ***: p-value ≤ 0.05, p-value ≤ 0.01, p-value ≤ 0.001, respectively.

[2] Positive: tolerance to the final PVY populations is significantly higher than to the initial variant; Negative: tolerance to the final PVY populations is significantly lower than to the initial variant; NS: not significant.

**Table S5.** Generalized linear models (GLMs) of variables related to PVY evolution during the experimental evolution or to host plant tolerance. The response variables are the difference in systemic viral load between the final PVY populations and the initial variants ($\Delta W$) (GLMs 1 and 2), the systemic viral load of final populations ($W_f$) (GLMs 3 to 7), the virulence of final populations ($VIR_f$) (GLMs 8 to 10) and the difference in plant tolerance to final and initial PVY ($\Delta TOL$) (GLMs 11 to 13). The explanatory variables correspond to the fitness or virulence of the initial variants ($W_i$ and $VIR_i$, respectively), the estimated size of the bottleneck imposed by the host plant on the PVY population at the inoculation stage ($N_e$) and the dynamics of infection in inoculated leaves ($\mu$, $k$ and $s$; Fig. S1). For each response variable, the GLM with the lowest corrected Akaike information criterion (cAIC) that meets (or nearly meets) the Shapiro-Wilk test ($p \geq 0.028$) of normality of residuals is highlighted in bold.

| GLM number | GLM selected | Shapiro-Wilk (p-value) | cAIC | R-squared |
|---|---|---|---|---|
| **1** | $\boldsymbol{\Delta W \sim \mu + k}$ | **0.045** | **67.5** | **0.156** |
| 2 | $\Delta W \sim s$ | 0.0125 | 66.8 | 0.132 |
| **3** | $\boldsymbol{W_f \sim W_i + k}$ | **0.205** | **71.1** | **0.670** |
| 4 | $W_f \sim W_i + s$ | 0.009 | 69.0 | 0.682 |
| 5 | $W_f \sim N_e + k + s + N_e \times k$ | 0.047 | 73.9 | 0.682 |
| 6 | $W_f \sim N_e + \mu + k + N_e \times \mu$ | 0.047 | 73.9 | 0.682 |
| 7 | $W_f \sim W_i + \mu$ | 0.011 | 73.9 | 0.653 |
| **8** | $\boldsymbol{VIR_f \sim VIR_i + N_e}$ | **0.053** | **-96.6** | **0.481** |
| 9 | $VIR_f \sim N_e + \mu + N_e \times \mu$ | 0.002 | -95.0 | 0.489 |
| 10 | $VIR_f \sim N_e + s + N_e \times s$ | 0.002 | -95.0 | 0.488 |
| **11** | $\boldsymbol{\Delta TOL \sim k + s + k \times s}$ | **0.028** | **147.6** | **0.211** |
| 12 | $\Delta TOL \sim N_e$ | 0.040 | 149.2 | 0.117 |
| 13 | $\Delta TOL \sim N_e + \mu + s + N_e \times \mu$ | 0.003 | 147.7 | 0.246 |

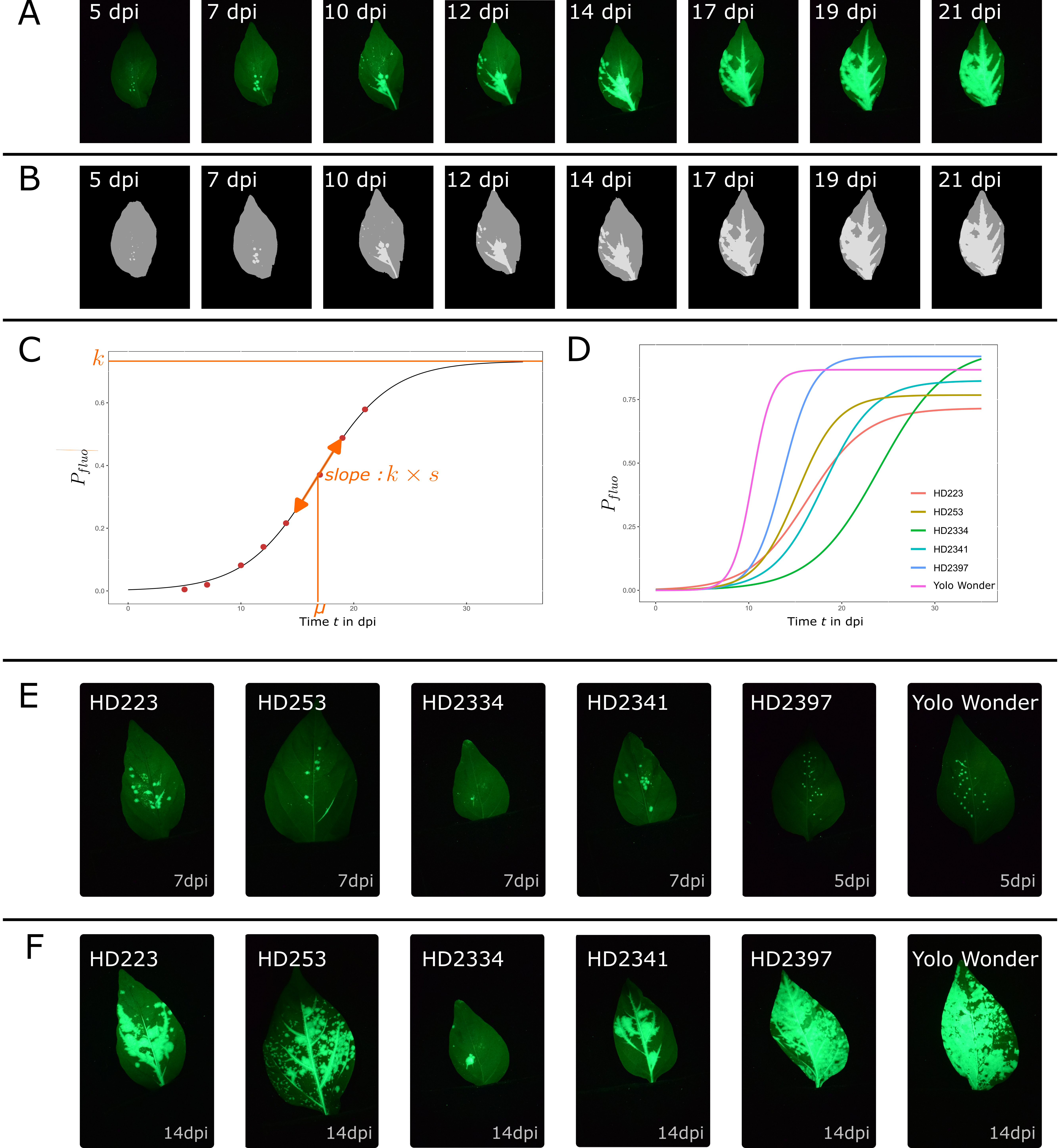

A
5 dpi
7 dpi
10 dpi
12 dpi
14 dpi
17 dpi
19 dpi
21 dpi
B
5 dpi
7 dpi
10 dpi
12 dpi
14 dpi
17 dpi
19 dpi
21 dpi
C
k
$P_{fluo}$
slope : $k \times s$
μ
Time t in dpi
D
$P_{fluo}$
HD223
HD253
HD2334
HD2341
HD2397
Yolo Wonder
Time t in dpi
E
HD223 7dpi
HD253 7dpi
HD2334 7dpi
HD2341 7dpi
HD2397 5dpi
Yolo Wonder 5dpi
F
HD223 14dpi
HD253 14dpi
HD2334 14dpi
HD2341 14dpi
HD2397 14dpi
Yolo Wonder 14dpi

**Figure S1.** Extraction of relevant parameters to assess PVY colonization dynamics of pepper leaves. Pepper accessions belonged to five DH lines and the Yolo Wonder susceptible control. **A**: Images taken five to 21 days post-inoculation (dpi) of pepper leaves inoculated with a GFP-tagged PVY variant. **B**: Result of a custom script to segment images into three areas: background (black), healthy leaf tissues (grey), and fluorescent infected tissues (white). **C**: Adjustment of the temporal evolution of the percentage of infected tissue ($P_{fluo}$) to the Verhulst model: $P_{fluo}(t) = \frac{k}{1+e^{-4s(t-\mu)}}$. $P_{fluo}$ was extracted from segmented images of each leaf. The model allows each curve to be described by three parameters: $\mu$ (the x-value at the inflection point of the curve, in dpi), $k$ (the value of the asymptote of the logistic curve) and $s$ (a parameter such that the slope at the inflection point is equal to $k \times s$). **D**: Plot of $P_{fluo}$ temporal evolution using means of $\mu$, $k$ and $s$ for each pepper line (Table S1). **E**: Photos of leaves at early stages of infection (5 or 7 dpi) enabling us to count the number of primary infection foci. **F**: Images of infected leaves characteristic of each pepper accession at 14 dpi.

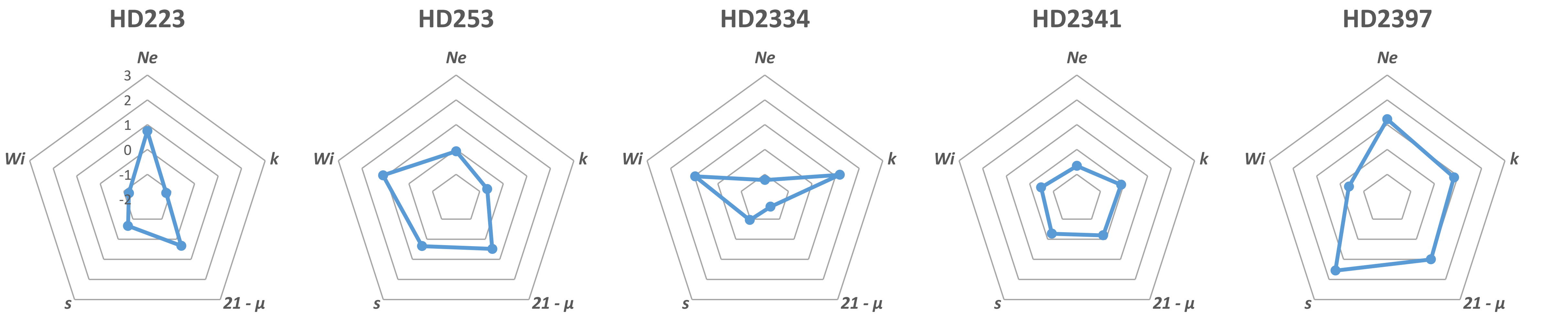


**Figure S2.** Radar diagrams of the susceptibility profile of pepper DH lines. The DH lines are presented according to five susceptibility traits to PVY: $N_e$ the number of primary infection foci, $W_i$ the systemic viral load, $\mu$, $k$ and $s$ corresponding to PVY colonization parameters of inoculated leaves (Fig. S1). As $\mu$ is a resistance trait, it was transformed into $(21 - \mu)$ to obtain a susceptibility trait since infection was recorded up to 21 dpi. Data were centered and reduced to assign a similar scale to all five traits. For HD223 and HD2397, $W_i$ values obtained with the two PVY variants K and GK were averaged.

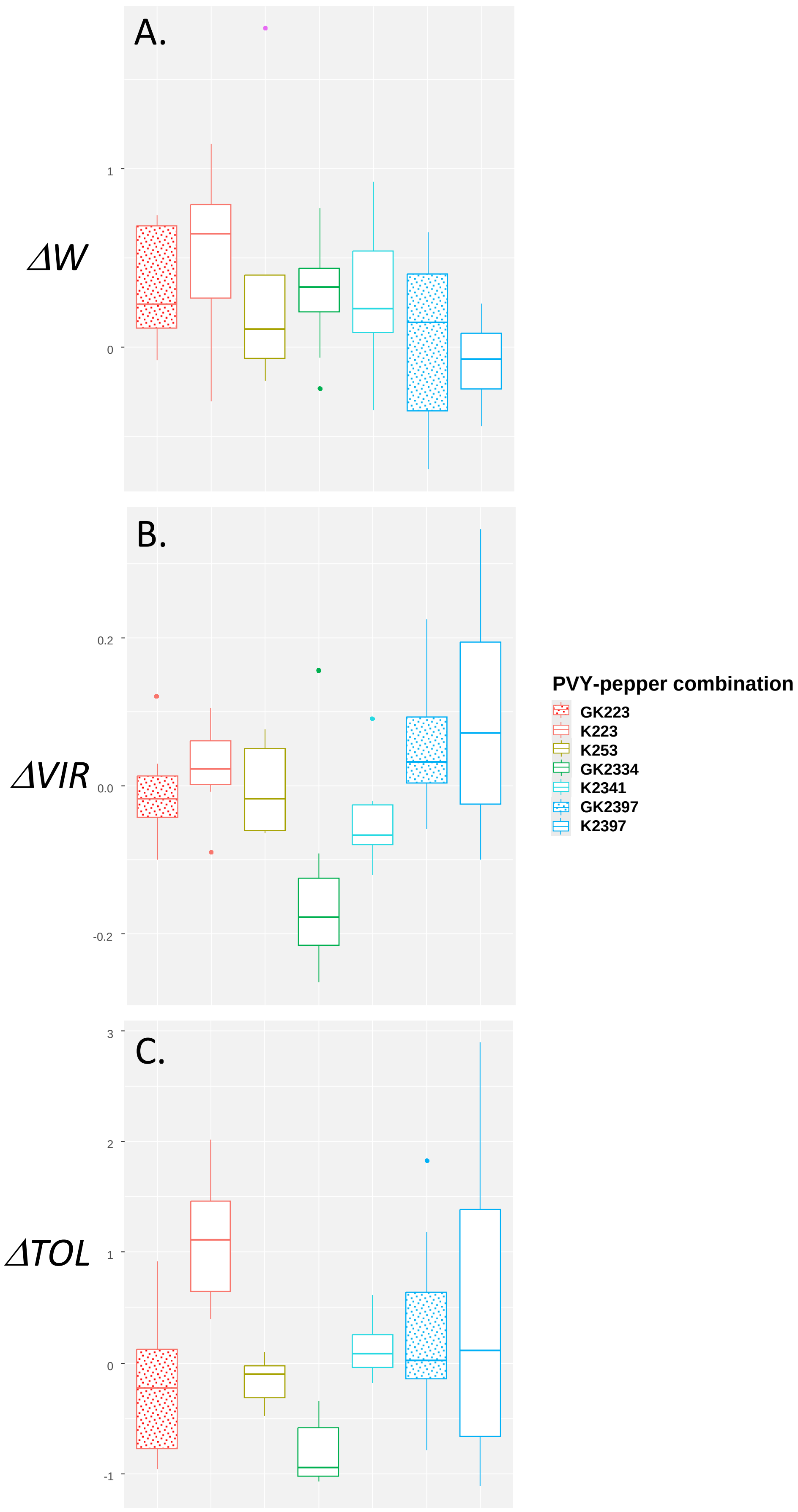


**Figure S3.** Differences in systemic viral load (A) and virulence (B) between final PVY populations and initial variants and difference between plant tolerance to final PVY populations and initial variants (C). Differences are shown for each combination of pepper DH line and virus variant.
